# Activation of Smurf E3 Ligase Promoted by Smoothened Regulates Hedgehog Signaling through Targeting Patched Turnover

**DOI:** 10.1371/journal.pbio.1001721

**Published:** 2013-11-26

**Authors:** Shoujun Huang, Zhao Zhang, Chunxia Zhang, Xiangdong Lv, Xiudeng Zheng, Zhenping Chen, Liwei Sun, Hailong Wang, Yuanxiang Zhu, Jing Zhang, Shuyan Yang, Yi Lu, Qinmiao Sun, Yi Tao, Feng Liu, Yun Zhao, Dahua Chen

**Affiliations:** 1 State Key Laboratory of Reproductive Biology, Institute of Zoology, Chinese Academy of Sciences, Beijing, China; 2 State Key Laboratory of Cell Biology, Institute of Biochemistry and Cell Biology, Shanghai Institutes for Biological Sciences, Chinese Academy of Sciences, Shanghai, China; 3 State Key Laboratory of Biomembrane and Membrane Biotechnology, Chinese Academy of Sciences, Beijing, China; 4 Centre for Computational and Evolutionary Biology, Chinese Academy of Sciences, Beijing, China; 5 Key Laboratory of Animal Ecology, Conservational Biology, Institute of Zoology, Chinese Academy of Sciences, Beijing, China; Stanford University, United States of America

## Abstract

Hedgehog signaling plays conserved roles in controlling embryonic development; its dysregulation has been implicated in many human diseases including cancers. Hedgehog signaling has an unusual reception system consisting of two transmembrane proteins, Patched receptor and Smoothened signal transducer. Although activation of Smoothened and its downstream signal transduction have been intensively studied, less is known about how Patched receptor is regulated, and particularly how this regulation contributes to appropriate Hedgehog signal transduction. Here we identified a novel role of Smurf E3 ligase in regulating Hedgehog signaling by controlling Patched ubiquitination and turnover. Moreover, we showed that Smurf-mediated Patched ubiquitination depends on Smo activity in wing discs. Mechanistically, we found that Smo interacts with Smurf and promotes it to mediate Patched ubiquitination by targeting the K1261 site in Ptc. The further mathematic modeling analysis reveals that a bidirectional control of activation of Smo involving Smurf and Patched is important for signal-receiving cells to precisely interpret external signals, thereby maintaining Hedgehog signaling reliability. Finally, our data revealed an evolutionarily conserved role of Smurf proteins in controlling Hh signaling by targeting Ptc during development.

## Abstract 2

Protein turnover of Patched, the Hedgehog receptor and key negative regulator of Hedgehog signaling, is controlled by the ubiquitin E3 ligase, Smurf, in a manner that depends on activation of signal transducer, Smoothened.

## Author Summary

Hedgehog (Hh) signaling is a pathway renowned for its roles in controlling embryonic development and tumorigenesis. Signaling via this pathway proceeds when Hh ligands bind to the receptor Patched (Ptc), thereby preventing Ptc from inhibiting the signal transducer, Smoothened (Smo), and thus allowing Smo to accumulate on the cell surface where it becomes activated and promotes downstream signal transduction. In the absence of Hh ligands, Ptc inhibits Smo and is a key negative regulator of Hh signaling. In this study, we investigate how protein turnover of Ptc is controlled to ensure tight regulation of Hh signaling. Using *Drosophila* as a model system, we provide biochemical and genetic evidence to show that the E3 ligase, Smurf, directly controls Ptc protein turnover in developing wing discs. Moreover, we found that Smurf mediates Ptc degradation in a manner that depends on Smo signaling activity: activated Smo forms a complex with Smurf to preferentially promote degradation of the ligand-unbound Ptc receptor. Using mathematic modeling we reveal that the control of Smo activation by the opposing activities of Smurf and Ptc, is important for cells receiving the Hh signal to precisely interpret and relay external signals. We show that this control mechanism is also active in vertebrates with evidence that zebrafish Smurf proteins target Ptc1 protein for degradation to control late somitogenesis during zebrafish embryogenesis.

## Introduction

Hedgehog (Hh) signaling is evolutionarily conserved and is essential for patterning of organs of both invertebrates and vertebrates [1],[2]. Dysregulation of Hh signaling activity leads to developmental abnormalities and cancers [3]. In *Drosophila*, genetic and biochemical evidence revealed that two multi-span transmembrane proteins, Patched (Ptc, 12-span) and Smoothened (Smo, 7-span), serve as a reception system for Hh signal transduction in Hh-receiving cells [4],[5]. In the absence of Hh ligands, Ptc inhibits Smo activity, thereby blocking Hh signaling transduction, while in the presence of Hh, the Hh ligands, in concert with its co-factor iHog, physically interacts with Ptc and alleviates its inhibition on Smo, resulting in Smo accumulation on the cell surface and a conformational switch of Smo to an active form that regulates the distinct downstream target genes in an Hh concentration-dependent manner [6]–[12].

In Hh signaling, the mechanisms by which Smo activation leads to downstream target gene expression are generally understood [2],[3]. However, less is known about the regulation of the Hh signaling receptor Ptc. Given that *ptc* is a direct target of the Hh pathway and that Ptc itself negatively regulates Hh signaling [9], Ptc expression must be tightly controlled to ensure proper Hh signal transduction. Previous studies have also shown that endogenous Ptc protein in Hh-receiving cells exhibits both plasma membrane and punctate-distribution patterns upon Hh ligand stimulation [13],[14], suggesting that Hh signal potentially promotes Ptc turnover. However, the molecular mechanism underlying Ptc degradation in response to Hh signal remains largely unknown.

Protein turnover mediated by ubiquitin modification plays important roles in the regulation of numerous cellular processes during development. The enzymatic reaction of protein ubiquitination is a highly ordered multi-step process involving three classes of enzymes, including ubiquitin-activating enzymes (E1s), ubiquitin-conjugating enzymes (E2s), and ubiquitin ligases (E3s) [15],[16]. E3 ubiquitin ligases are crucial in the ubiquitin conjugation cascade because of their roles in the recruitment of ubiquitin-loaded E2s and their selective recognition of target proteins. Generally, the E3 ubiquitin ligases are classified into three subfamilies: the really interesting new gene (RING) finger domain containing E3s, the homologous to E6-AP carboxyl terminus (HECT) domain containing E3s, and the U box E3s [15],[17]. Previous studies have shown that Neural precursor cell expressed, developmentally downregulated 4 (Nedd4), one member of the C2-WW-HECT family proteins, could physically associate with the Ptc protein [13],[18]; however, whether the Nedd4 is involved in the regulation of Hh signaling activity through its interaction with Ptc remains unknown. Smad ubiquitin regulatory factor (Smurf) proteins are other members of the C2-WW-HECT E3 family of proteins that contain typical WW and HECT domains. Smurf proteins (including Smurf1 and Smurf2 in mammals) were originally identified as an E3 ubiquitin ligase for the degradation of R-Smad proteins and type I receptors to negatively regulate TGFβ/BMP signal [19]–[25]. Recently, Smurfs have also been shown to regulate cell motility by targeting RhoA for ubiquitin-mediated degradation [26],[27], and are involved in the non-canonical Wnt signaling to regulate planar cell polarity by degrading the PCP core component, Prickle1 protein [28]. We recently uncovered that Smurf functions in concert with Fused, a serine/threonine kinase that regulates Hedgehog signaling, to degrade the BMP type I receptor Tkv allowing for bam expression in differentiating cystoblast cells, thereby determining the fate of *Drosophila* germline stem cells [22],[24]. These studies revealed that Smurf proteins have diverse biological functions through regulating multiple signal pathways in different cellular contexts. In this study, we identified a novel role of Smurf E3 ligase in the regulation of Hh signaling by directly controlling Ptc protein turnover. Moreover, we found that Smurf mediates Ptc degradation in a manner that depends on Smo signaling activity. These findings revealed a novel mechanism by which an Hh signaling-dependent bidirectional control mechanism involving Ptc and Smurf is important for signal-receiving cells to precisely interpret external signal, thereby maintaining the reliability of Hh signaling transduction. Finally, we provided evidence that zebrafish Smurf proteins also modulate Hh signaling by controlling Ptc1 protein degradation to regulate late somitogenesis during zebrafish embryogenesis. Thus, our data support the conclusion that Smurf proteins have an evolutionarily conserved role in controlling Hh signaling during development.

## Results

### Identification of Ptc as a Smurf-Interacting Protein

**Figure 1:**
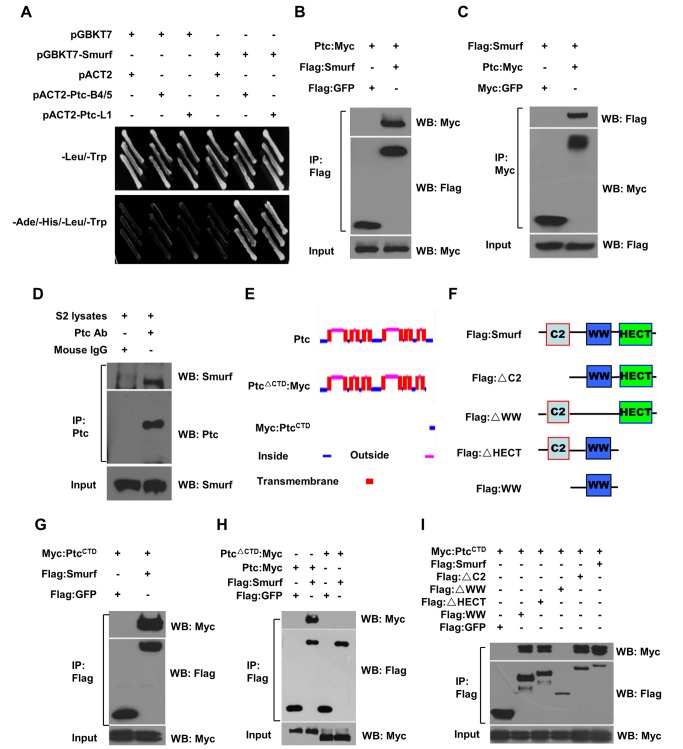
Identification of Ptc as a Smurf-interacting protein. (A) A yeast two-hybrid screen was performed that identified Ptc as a Smurf-interacting protein. The AH109 yeast strain was transformed with the indicated plasmids and plated at permissive (−Leu/−Trp) and restrictive (−Ade/−His/−Leu/−Trp) medium. Full-length *smurf* were cloned into the bait vector, pGBKT7, as the bait for library screen; and pACT2 is the prey vector. B4/5 (aa 1174–1286) and L1 (aa 1198–1286) were two independent Ptc clones from the screen. (B and C) S2 cells were transfected with combinations of DNA constructs as indicated. After 48 h transfection, lysates from transfected S2 cells were immunoprecipitated with anti-Flag M2 affinity gel (B) or anti-Myc affinity gel (C). Western blots were performed to analyze the presence of Flag- or Myc-tagged proteins. (D) S2 cell lysates were immunoprecipitated with mouse anti-Ptc antibody or control mouse IgG. Western blots were performed to analyze the presence of Ptc or Smurf proteins. (E and F) Schematic drawings of Ptc (E) and Smurf (F) and their deletion constructs. (G–I) S2 cells were transfected with combinations of DNA constructs as indicated. After 48 h transfection, lysates from transfected S2 cells were immunoprecipitated with anti-Flag M2 affinity gel. Western blots were performed to analyze the presence of Flag- or Myc-tagged proteins.

To explore novel biological functions of Smurf, we searched for Smurf-interacting partners by performing a yeast two-hybrid screen using the full-length *Drosophila* Smurf as bait. From this screen, we found three positive clones that encode two different C-terminal fragments of the *Drosophila* Ptc protein, Ptc-B4/5 and Ptc-L1 (Figure 1A; Table S1), indicating that Ptc could be a potential candidate as a Smurf-interacting partner. To confirm the interaction between Ptc and Smurf proteins, we then performed co-immunoprecipitation experiments, and found that Flag-tagged Smurf and Myc-tagged Ptc co-immunoprecipitated with each other in transfected S2 cells (Figure 1B and 1C). Consistent with this, endogenous Smurf protein could be co-immunoprecipitated by anti-Ptc antibody (Figure 1D), revealing that Smurf and Ptc proteins are able to form a complex together in S2 cells. In agreement with the yeast-two-hybrid assay, domain-mapping analysis of Ptc revealed that the C-terminal tail was necessary and sufficient for Ptc to interact with Smurf (Figure 1E, 1G, and 1H). To further define the essential domain(s) in Smurf required for its interaction with Ptc, we employed a series of previously described truncated forms of Smurf (Figure 1F) [24] in co-immunoprecipitation experiments. As shown in Figure 1I, while the C2 and HECT domains of Smurf were dispensable for its interaction with Ptc, the WW domains were essential for Smurf to form a complex with Ptc, suggesting that Smurf interacts with Ptc through its WW domains.

### Smurf Promotes Ptc Ubiquitination and Degradation through Its C-Tail

**Figure 2:**
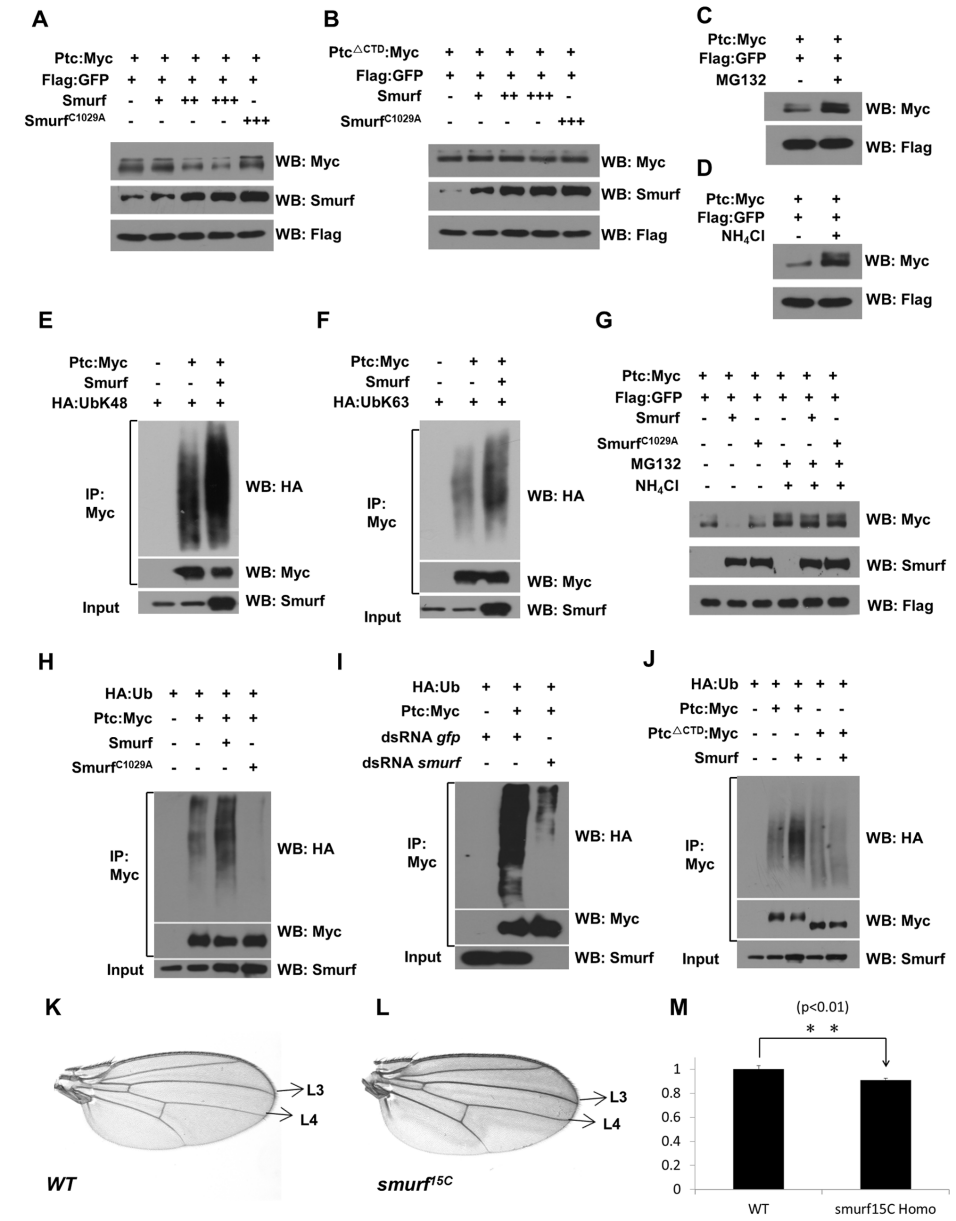
Smurf promotes Ptc ubiquitination and degradation through its C-tail. (A and B) S2 cells were transfected with combinations of DNA constructs as indicated. After 48 h transfection, lysates from transfected S2 cells were used to quantify Ptc:Myc levels (A) and Ptc^ΔCTD^:Myc (B) by Western blots. (C and D) S2 cells were transfected with combinations of DNA constructs as indicated. After 48 h transfection, S2 cells were treated with MG132 at a final concentration of 50 µM (C) or NH_4_Cl at a concentration of 50 mM (D) for 4 h, lysates from transfected S2 cells were used to quantify the levels of Ptc:Myc by Western blots. (E) S2 cells were transfected with combinations of DNA constructs as indicated. After 48 h transfection, S2 cells were treated with MG132 at a final concentration of 50 µM for 4 h. Lysates from transfected S2 cells were immunoprecipitated with mouse anti-Myc affinity gel. Western blots were performed to analyze the presence of indicated proteins and levels of ubiquitination (UbK48). (F) S2 cells were transfected with combinations of DNA constructs as indicated. After 48 h transfection, S2 cells were treated with NH_4_Cl at a concentration of 50 mM for 4 h. Lysates from transfected S2 cells were immunoprecipitated with mouse anti-Myc affinity gel. Western blots were performed to analyze the presence of indicated proteins and levels of ubiquitination (UbK63). (G) S2 cells were transfected with combinations of DNA constructs as indicated. After 48 h transfection, S2 cells were treated with both MG132 and NH_4_Cl for 4 h; lysates from transfected S2 cells were used to quantify levels of Ptc:Myc by Western blots. (H) S2 cells were transfected with combinations of DNA constructs as indicated. After 48 h transfection, S2 cells were treated with MG132 and NH_4_Cl for 4 h. Lysates from transfected S2 cells were immunoprecipitated with mouse anti-Myc affinity gel. Western blots were performed to analyze the presence of indicated proteins and levels of ubiquitination. (I) S2 cells were treated with dsRNA against *gfp* or *smurf* by starvation method. After 48 h treatment, cells were transfected with combinations of DNA constructs as indicated for another 48 h. S2 cells were treated with MG132 and NH_4_Cl for 4 h, lysates from transfected S2 cells were immunoprecipitated with mouse anti-Myc affinity gel. Western blots were performed to analyze the presence of indicated proteins and levels of ubiquitination. (J) S2 cells were transfected with combinations of DNA constructs as indicated. After 48 h transfection, S2 cells were treated with MG132 and NH_4_Cl for 4 h. Lysates from transfected S2 cells were immunoprecipitated with mouse anti-Myc affinity gel. Western blots were performed to analyze the presence of indicated proteins and levels of ubiquitination. (K and L) Compared with the adult wing of a wild-type fly (K), *smurf* mutant causes reduction of the area of the intervein region between L3 and L4 (L). (M) Adult wings were quantified by measuring the area between L3 and L4 space and the whole wing (*n* = 18). Results were expressed as the ratio of the two areas.

WW domains are typical domains in C2-WW-HECT subfamily E3 ligases for substrate recognition through their interaction with a proline-rich sequence (PPXY motif) in substrate proteins [15],[29]. Given that the C-terminal region of Ptc contains a consensus PPXY motif that has been shown to be required for the Ptc interaction with Nedd4, the other member of C2-WW-HECT subfamily E3 ligases [13],[18],[30], and that mutations of PPXY in the C-terminal region of Ptc abolished the Ptc-Smurf interaction, as indicated in the GST pull-down and yeast-two-hybrid assays (Figures S1A and S8A), we reasoned that Ptc might be a strong candidate as a substrate of Smurf and determined whether Smurf regulates Ptc stability in S2 cell cultures. As shown in Figure 2A and 2B, overexpression of Smurf apparently downregulated the full-length Ptc, but not mutant Ptc (Ptc^ΔCTD^) that lacks the C-tail of Ptc, which showed more stability than the full-length Ptc (Figure S1B). Interestingly, the level of Ptc protein could be significantly increased when cells were treated with either MG132, a proteasome inhibitor, or NH_4_Cl, a lysosome inhibitor (Figure 2C and 2D), and Smurf promotes both Ub K48-linked and Ub K63-linked ubiquitination of Ptc, as indicated by multiple co-immunoprecipitation experiments followed by Western blot assays (Figures 2E, 2F, and S1C–S1F), suggesting the stability of Ptc is regulated through both proteasome and lysosome pathways. Consistent with this observation, the downregulation of the full-length of Ptc protein mediated by co-expression of Smurf was markedly blocked by treatments with both MG132 and NH_4_Cl (Figure 2G). Collectively, our data argue that Smurf promotes Ptc degradation through its C-tail in cell cultures.

We then determined whether Smurf promotes Ptc ubiquitination by performing ubiquitination assays in S2 cells according to the methods described previously [31]–[33]. As shown in ubiquitination assays, overexpression of the wild-type form of Smurf significantly increased the ubiquitin conjugation of full-length Ptc or the C-tail of Ptc, but the catalytic dead form of Smurf (Smurf^C1029A^) [19] did not (Figures 2H and S2A–S2E). We noted that overexpression of Smurf^C1029A^ reduced ubiquitination of Ptc, suggesting its dominant negative role in regulating Ptc ubiquitination in S2 cells, likely due to the fact that Smurf^C1029A^ interacted with Ptc in cultured cells, and thus competitively inhibited endogenous Smurf function (Figure S2F). Consistent with these observations, we found that knockdown of Smurf markedly reduced the full-length Ptc ubiquitination in S2 cells (Figure 2I). To ask whether the C-tail of Ptc is responsible for its ubiquitination mediated by Smurf, we co-overexpressed Ptc^ΔCTD^ and Smurf in S2 cells. As shown in Figure 2J, this truncated form of Ptc was apparently resistant to ubiquitination by Smurf E3 ligase. In agreement with these findings, the *in vitro* ubiquitination assays revealed that the C-tail of Ptc could be directly ubiquitinated by Smurf E3 ligase (Figure S2G). Taken together, our results support that Smurf promotes Ptc ubiquitination and degradation through its C-tail.

### Smurf Regulates Hh Signaling Pathway by Controlling Ptc Turnover *In Vivo*

**Figure 3:**
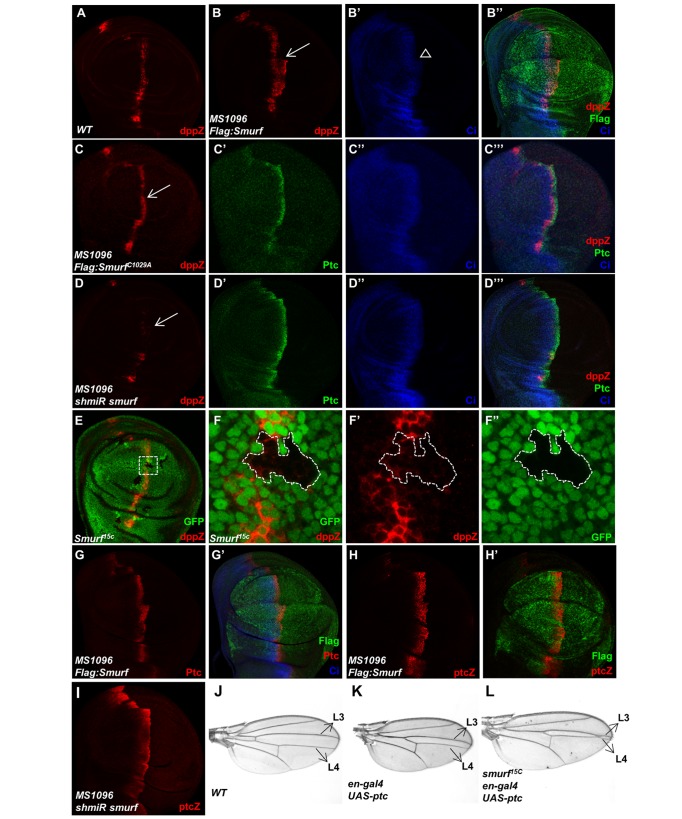
Smurf positively regulates Hh signaling pathway in wing discs. (A) Wild-type (WT) wing discs carrying *dpp-LacZ* (*dppZ*) reporter were immunostained with anti-β Gal antibody to show the expression of *dppZ* (red). (B–B″) Wing discs expressing Flag:Smurf driven by *MS1096-gal4* were immunostained to show the expression of *dppZ* (red), Ci (blue), and Flag (green). (C–C′″) Wing discs expressing Flag:Smurf^C1029A^ driven by *MS1096-gal4* were immunostained to show the expression of *dppZ* (red), Ptc (green), and Ci (blue). (D–D′″) Wing discs expressing shmiR *smurf* driven by *MS1096-gal4* were immunostained to show the expression of *dppZ* (red), Ptc (green), and Ci (blue). (E and F–F″) Low (E) and high (F–F″) magnification views of a *smurf^15c^* clone at A/P boundary. Immunostaining was performed to show the expression of *dppZ* (red) and GFP (green). *Smurf^15c^* clones are recognized by the lack of GFP. (G and G′) Wing discs expressing Flag:Smurf driven by *MS1096-gal4* were immunostained with Ptc antibody to show the expression of Ptc (red) and Flag (green). (H and H′) Wing discs expressing Flag:Smurf driven by *MS1096-gal4* were immunostained to show the expression of *ptcZ* (red) and Flag (green). (I) Wing discs expressing shmiR *smurf* driven by *MS1096-gal4* were immunostained to show the expression of *ptcZ* (red). (J–L) comparison of adult wing phenotype from wild-type flies (J), UAS-ptc overexpression driven by *en-gal4* (K), and UAS-ptc overexpression driven by *en-gal4* in *smurf^15C^* homozygous background (L).

To determine the biological link between Smurf and Hh signaling, we used *Drosophila* wing discs and adult wings as an assay system to assess the *in vivo* role of *smurf* in Hh signaling during development. In wing discs, Hh produced in the posterior compartment cells induces the anterior compartment cells adjacent to the A/P boundary to express *dpp* and *ptc*, which respond to low levels and higher levels of Hh activity, respectively. It has been documented that, while activation of Hh signaling is essential for the proper patterning of the intervein region between L3 and L4 of adult wing, downregulation of Hh signaling causes the reduction of the intervein region between L3 and L4 [34]. To test whether *smurf* is involved in the regulation of Hh signaling, we first measured the area between L3 and L4 space and the whole wing in wild type and *smurf* mutants. As shown in Figure 2K–2M, loss of *smurf* led to a slight but evident reduction (*n* = 18, *p*<0.01) of the intervein region between L3 and L4, compared to the wild-type control, suggesting that *smurf* might function as a positive modulator in Hh signaling pathway. To confirm this observation, we then overexpressed Flag-tagged Smurf or Flag-tagged Smurf^C1029A^ in the wing discs using the *MS1096-gal4*, a driver that has higher activity in the dorsal compartment of wing discs, and measured the expression levels of Ci and *dpp-lacZ*, the responsive reporter to the low level of Hh signaling. As shown in Figure 3A, 3B–3B″, and 3C–3C′″, overexpression of the wild-type form of Smurf, rather than Smurf^C1029A^, was able to not only increase expression of *dpp-lacZ*, but also slightly expanded the domain of both Ci and *dpp-lacZ* toward the P compartment. Similar results were obtained when the *ap-gal4*, a dorsal compartment specific driver, was employed (Figure S3A–S3A″, S3B–S3B″, and S3C–S3C″). We then expressed *smurf* RNAi transgene, P{*uasp-shmiR-smurf*} [35], to specifically knockdown *smurf* in wing discs using the *MS1096-gal4* or the *ap-gal4* driver, and found that inactivation of *smurf* decreased expression of *dpp-lacZ* (Figures 3D–3D′″ and S3C–S3C″). To further confirm these observations, we then performed the mosaic clonal analysis. Consistently, *dpp-lacZ* expression was significantly downregulated in *smurf* mutant cell clones located in the A/P boundary (Figure 3E and 3F–3F″). Taken together, our findings suggest that Smurf functions as a positive regulator in the Hh signaling pathway in wing discs.

We noted that overexpression of *smurf* by *MS1096-gal4* driver markedly increased *dpp-lacZ* expression, but did not apparently affect the levels of both Ptc protein and *ptc-lacZ*, which responds to a higher level of Hh signaling (Figure 3G–3H′). Accordingly, knockdown of *smurf* did not dramatically affect the global levels of Ptc protein either (Figure 3D′). One possibility could be that *ptc* transcripts and Ptc protein can compensate for each other, owing to the negatively regulatory role of Ptc protein in Hh signaling. To validate this issue, we blocked new Ptc protein synthesis from Ptc transcripts by pre-treatment of the discs with the protein synthesis inhibitor cycloheximide (CHX), and then assessed whether Smurf controls the Ptc protein turnover. As shown in Figure S3D–S3K, after the CHX treatment, overexpression of *smurf* by the *MS1096-gal4* greatly reduced the Ptc protein expression level, whereas knockdown of *smurf* evidently increased Ptc levels, compared to the discs without the CHX treatment. To confirm these findings, we then expressed the Ptc by the *en-gal4* driver in smurf mutants. As shown in Figure 3J–3L, expression of Ptc^WT^ led to much more severe wing phenotype in *smurf* mutants than that in wild-type background. These results reveal that Smurf positively regulates Hh signaling through controlling Ptc turnover.

### Activation of Smo Promotes Smurf-Mediated Ptc Ubiquitination

**Figure 4:**
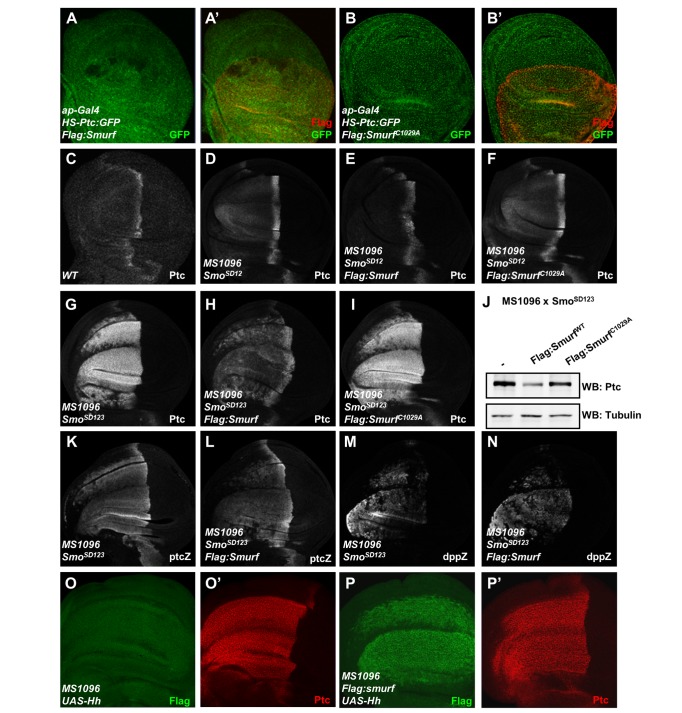
Smurf regulates Hh signaling by controlling Ptc turnover. (A–A′ and B–B′) Wing discs expressing Ptc:GFP fusion protein ubiquitously expressed by heat shock promoter, which also expressed Flag-Smurf or Flag-Smurf^C1029A^ driven by *ap-gal4*, were immunostained to show the intensity of GFP (green) and Flag (red). (C) Wild-type control discs were immunostained with anti-Ptc antibody to show Ptc protein expression. (D) Wing discs expressing Smo^SD12^ driven by *MS1096-gal4* were immunostained to show Ptc protein expression levels. (E and F) Wing discs expressing Smo^SD12^ and Flag:Smurf (E) or Flag:Smurf^C1029A^ (F) driven by *MS1096-gal4* were immunostained to show Ptc protein expression levels. (G) Wing discs expressing Smo^SD123^ driven by *MS1096-gal4* were immunostained to show Ptc protein expression. (H and I) Wing discs expressing Smo^SD123^ and Flag:Smurf (H) or Flag:Smurf^C1029A^ (I) driven by *MS1096-gal4* were immunostained to show Ptc protein expression. (J) Wing discs with indicated genotypes were used for Western blots to show levels of Ptc protein; α-tubulin was used as loading control. (K) Wing discs expressing Smo^SD123^ driven by *MS1096-gal4* were immunostained with anti-β-Gal antibody to show *ptcZ* expression. (L) Wing discs expressing Smo^SD123^ and Flag:Smurf driven by *MS1096-gal4* were immunostained to show *ptcZ* expression. (M) Wing discs expressing Smo^SD123^ driven by *MS1096-gal4* were immunostained with anti-β-Gal antibody to show *dppZ* expression. (N) Wing discs expressing Smo^SD123^ and Flag:Smurf driven by *MS1096-gal4* were immunostained to show *dppZ* expression. (O–O′) Wing discs expressing Hh driven by *MS1096-gal4* were immunostained to show Ptc expression. (P–P′) Wing discs expressing Hh and Flag:Smurf driven by *MS1096-gal4* were immunostained to show Ptc (red) and Flag (green) expression.

**Figure 5:**
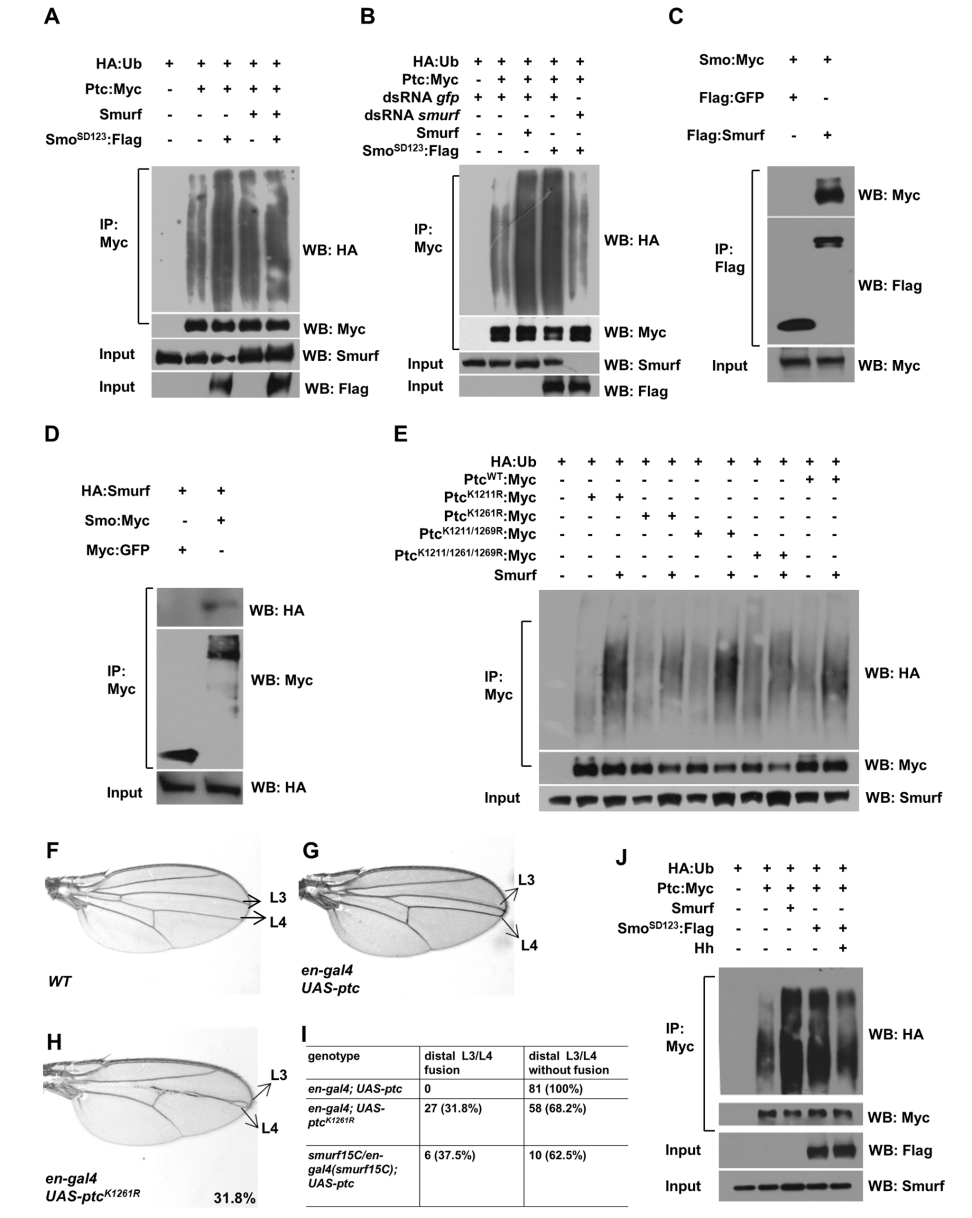
Smo promoted Smurf regulates Ptc ubiquitination through specific sites in Ptc C-tail. (A and B) S2 cells were transfected with combinations of DNA constructs as indicated. After 48 h transfection, S2 cells were treated with MG132 and NH_4_Cl for 4 h. Lysates from transfected S2 cells were immunoprecipitated with mouse anti-Myc affinity gel. Western blots were performed to analyze the presence of indicated proteins and levels of ubiquitination. (C and D) S2 cells were transfected with combinations of DNA constructs as indicated. After 36 h transfection, cells were treated with Hh medium for another 12 h to activate Hh signaling, lysates from transfected S2 cells were immunoprecipitated with anti-Flag M2 affinity gel (C) or anti-Myc affinity gel (D). (E) S2 cells were transfected with combinations of DNA constructs as indicated. After 48 h transfection, S2 cells were treated with MG132 and NH_4_Cl for 4 h. Lysates from transfected S2 cells were immunoprecipitated with mouse anti-Myc affinity gel. Western blots were performed to analyze the presence of indicated proteins and levels of ubiquitination. (F–H) Comparison of adult wing phenotype from wild-type flies (F), *UAS-ptc* overexpression driven by *en-gal4* (G), and *UAS-ptc^K1261R^* overexpression driven by *en-gal4* (H). (I) Phenotypic classification and statistics of flies with indicated genotypes, the ratio of fused veins between distal L3 and L4 in adult wing was measured in each genotype. (J) S2 cells were transfected with combinations of DNA constructs as indicated. After 48 h transfection, S2 cells were treated with MG132 and NH_4_Cl for 4 h. Hh medium was collected from a pMT-Hh stable transfected S2 cell line, and treated S2 cells for 12 h before cell harvesting. Lysates from transfected S2 cells were immunoprecipitated with mouse anti-Myc affinity gel. Western blots were performed to analyze the presence of indicated proteins and levels of ubiquitination.

Given that the function of Smurf that targets Ptc is restricted to the A/P boundary cells, which exhibit high levels of Hh signaling activity, we reasoned that Smurf might regulate Ptc protein turnover via responding to the high levels of Hh signaling activity. However, a recent study proposed that Ptc degradation might depend on Ptc itself, since a high level of Ptc is also present in the A/P boundary cells [30]. To distinguish these two potential mechanisms by which Smurf mediates Ptc degradation, we employed the P{*hs-ptc:gfp*} transgene to overexpress Ptc:green fluorescent protein (GFP) in the wing discs by heat-shock treatment, which is independent of Hh signaling. As shown in Figure 4A–4A′ and 4B–4B′, overexpression of either the wild-type Smurf or Smurf^C1029A^ by the *ap-gal4* driver did not affect the expression of Ptc:GFP induced by heat-shock treatment in the wing discs, suggesting that expression of Ptc itself is not sufficient to induce Smurf-mediated Ptc degradation. To test whether Smurf could regulate Ptc turnover in response to Hh signaling, we next established an ectopic Hh signaling activation system by overexpression of the activated forms of Smo (Smo^SD12^ or Smo^SD123^) [11] in the A-compartment of wing discs. As shown in Figure 4C–4J, overexpression of either Smo^SD12^ or Smo^SD123^ was sufficient to upregulate Ptc expression (Figure 4D and 4G). In particular, the high level of Ptc in the A-compartment cells induced by Smo^SD123^ was comparable with that in the A/P boundary cells (Figure 4G). Given that Hh is absent in A-compartment cells away from A/P boundary and that activated Smo-induced Hh signaling is independent of Ptc expression, this system circumvents the compensatory feedback regulation between Ptc expression and Hh signaling in the A-compartment cells. We then probed whether Smurf degrades Ptc in the cells with ectopic Hh signaling activity. As shown in Figure 4E, 4F, 4H, and 4I, overexpression of Smurf but not Smurf^C1029A^ by the *MS1096-gal4* significantly downregulated the levels of Ptc protein ectopically induced by the expression of Smo^SD12^ or Smo^SD123^ in A compartment cells (Figure 4D and 4H). Moreover, we found that overexpression of Smurf by the *MS1096-gal4* did not affect Hh signaling activity induced by the activated Smo, as indicated by the fact that the expression levels of *ptc-lacZ* or *dpp-lacZ* were not evidently changed in Figure 4K–4N, although overexpression of Smurf slightly reduced Smo^SD123^ levels (unpublished data). Thus, activation of Smo is sufficient to promote Ptc protein degradation *in vivo*. To test whether the Smurf-mediated Ptc degradation is attributed to activation of Smo downstream signaling, we performed genetic assays by overexpression of constitutively activated form of Ci (Ci^103^) in the wing disc [8],[11]. As shown in Figure S4A–S4D, overexpression of Smurf had no detectable effect on Ptc protein stability induced by Ci^103^ expression in wing discs. Thus, our results suggest that Smurf-mediated Ptc protein degradation largely depends on activation of Smo itself but not its downstream signaling. To gain biochemical evidence, we then performed the ubiquitination assay in S2 cells. As shown in Figure 5A and 5B, overexpression of Smo^SD123^ significantly increased the Ptc ubiquitination by Smurf; however, knockdown of Smurf markedly reduced Ptc ubiquitination promoted by overexpression of Smo^SD123^. Thus, our findings further confirm that activated Smo promotes Smurf-mediated Ptc ubiquitination and degradation.

### Smo Interacts with Smurf and Promotes Ptc Ubiquitination in a Site-Specific Manner

Having seen that Smo promotes Smurf-mediated Ptc ubiquitination, we then sought to investigate whether activated Smo and Smurf act in a common pathway to regulate Ptc ubiquitination by forming a complex. As shown in co-immunoprecipitation assays (Figures 5C, 5D, and S4E), Smurf could be associated with Smo through the C-tail of Smo, emphasizing that activated Smo promotes Smurf-mediated Ptc ubiquitination in a Smo activation-dependent manner. Since Smurf and Smo form a complex, we then asked whether Smurf regulates ubiquitination and stability of Smo in S2 cells and wing discs. As shown in Figure S4F and S4G, knockdown of *smurf* did not affect Smo ubiquitination and stability in S2 cells, and knockdown of *smurf* in wing disc clone cells caused no apparent change of endogenous Smo protein expression, when compared to the neighbor wild-type cells (Figure S4H–S4H″ and S4I–S4I′″). Thus, our results suggest that Smurf regulates Hh signaling primarily through targeting Ptc, but not Smo, for ubiquitination.

To further understand the molecular basis of how Ptc is ubiquitinated through its C-tail, we sought to search for the specific site(s) in Ptc C-tail that respond to the Smurf E3 ligase. We thus generated a series of mutant forms of Ptc, in which the K sites in the Ptc C-tail were mutated to R individually or in combination. As shown in ubiquitination assays, both Ptc^K1261R^ and Ptc^K1211R, K1261R, K1269R^ mutants were evidently resistant to being ubiquitinated by Smurf (Figures 5E and S5A), suggesting the K1261 site is specifically regulated by Smurf in S2 cell cultures. To determine the biological function of the K1261 site of Ptc, we generated a ubiquitin-resistant form of the Ptc transgene line, P{*UAS-Ptc^K1261R^*}, in which the K1261 site was mutated to R. As shown in Figure 5F–5I, expression of Ptc^K1261R^ by the *en-gal4* driver in the posterior compartment of wing disc caused extreme reductions in the intervein region between L3 and L4 veins, whereas expression of Ptc^WT^ led to a less severe wing phenotype. In support of this idea that the greater severity of the Ptc^K1261R^ phenotypes was attributed to the resistance of Ptc^K1261R^ to ubiquitination by Smurf E3 ligase, expression of Ptc^WT^ by the *en-gal4* driver in the *smurf* mutants caused much more severe intervein phenotype than that in wild type (Figures 3J–3L and S5A), demonstrating the biological importance of the K1261 site of Ptc in the regulation of Hh signaling. The previous study has shown that residues (T1260, T1263, T1265) are vital for Ptc trimerization. Interestingly, the K1261 we identified is very close to these residues, and disruption of these residues resulted in a similar phenotype to that in the wing expressing Ptc^K1261R^ [13], raising a possibility that K1261 might contribute to the Ptc trimerization. To test this, we used the native gel and performed Western blot assays, and found that the K1261R mutation had no effect on the Ptc trimerization (Figure S5B). Taken together, our results strongly argue that Smurf regulates Ptc ubiquitination through its K1261 site *in vivo*.

### Smurf Has a Preference for Targeting Ligand-Unbound Ptc

It has been proposed that the ratio of Hh ligand bound to unbound Ptc protein is important in determining Hh signaling activity in *Drosophila* wing discs [36]. However, the molecular mechanism of how Ptc degradation is regulated remains elusive. In this study, we found that Hh ligand-independent Smo activation is sufficient to promote Smurf-mediated Ptc ubiquitination and degradation. Interestingly, as shown in Figure 4D and 4H, we noted that downregulation of Ptc by Smurf in A compartment cells appears much more effective than that in the A/P boundary. Given that the Hh molecules are highly presented in A/P boundary but absent in A-compartment cells away from the A/P boundary, our findings thus raised a possibility that Smurf might have a preference for targeting unbound Ptc in wing discs. To test this, we performed ubiquitination experiments in S2 cells. S2 cells were co-transfected with Ptc, Smurf, and SmoSD^123^, and then treated with Hh-conditioned or control medium. As shown in Figure 5J, the levels of Ptc ubiquitination from cells with Hh treatment were apparently lower than that from control cells, suggesting that SmoSD^123^ promotes Smurf-mediated Ptc ubiquitination more efficiently in the absence of Hh. To further verify this data *in vivo*, we co-expressed Hh with or without Smurf in wing discs using *MS1096-gal4* driver. As shown in Figure 4O–4P′, overexpression of Hh resulted in high levels of Ptc expression in A-compartment cells, which were comparable with the A/P boundary cells. However, we found that, in contrast to the fact that Smurf overexpression significantly downregulated Smo^SD^-induced Ptc expression in A-compartment cells, compared with that in A/P boundary cells, overexpression of Smurf had no apparent effect on Hh ligand-induced Ptc expression in A-compartment cells, compared with that in A/P boundary cells. Thus, our data strongly argue that Smurf has a preference for targeting ligand-unbound Ptc.

### Dynamic Analysis of the Role of Smurf in the Hh Signaling Pathway in Wing Disc

How does the preferential targeting of ligand-unbound Ptc by Smurf contribute to Hh signaling transduction? The previous study proposed an elegant model by which the ratio of ligand bound to unbound Ptc protein is important in determining Hh signaling activity in *Drosophila* wing discs [36]. Our experimental data reveal that Smurf could control the ratio of ligand bound to unbound Ptc protein through preferentially targeting ligand-unbound Ptc in wing disc, thereby precisely determining Hh signaling. Additionally, this regulation apparently depends on Hh signaling, particularly promoted by activation of Smo.

**Figure 6:**
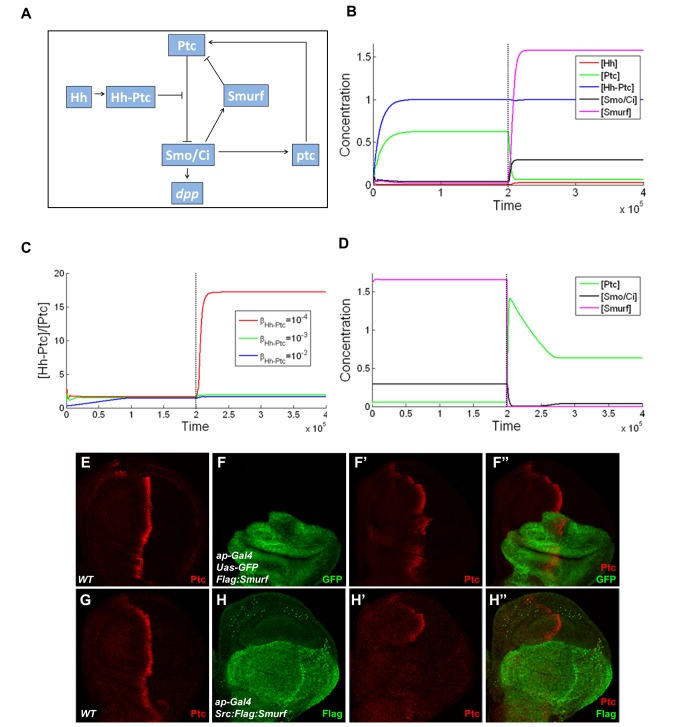
Bidirectional regulation and role of activated Smurf in the Hh signaling pathway. (A) A simplified Hh signaling pathway network for mathematic modeling. (B) The effects of over expression of activated Smurf on [Hh], [Ptc], [Hh_Ptc], [activated Smo/Ci]. On the left, the levels of [Hh], [Ptc], [Hh_Ptc], and [activated Smo/Ci] correspond to the low level of [activated Smurf], and, similarly, on the right, the levels of [Hh], [Ptc], [Hh_Ptc], and [activated Smo/Ci] correspond to the high level of [activated Smurf] (i.e., over expression of activated Smurf). (For the value of each parameter, see Table S1.) (C) The effect of over expression of activated Smurf on the ratio [Hh_Ptc]/[Ptc]. For all three values of *β_Hh_Ptc_*, the level of ratio [Hh_Ptc]/[Ptc] corresponding to the low activated Smurf is low (on the left). However, when the activated Smurf is over expressed, the level of ratio [Hh_Ptc]/[Ptc] corresponding to *β_Hh_Ptc_* = 10^−4^ is significantly high. (For the value of each parameter, see Table S1.) (D) The effects of the inhibition of activated Smurf on [Ptc] and [activated Smo/Ci]. When the expression of activated Smurf is inhibited, the levels of [Ptc] and [activated Smo/Ci] decrease immediately (on the right). (For the value of each parameter, see Table S1.) (E and F–F″) Wild-type control discs were immunostained with anti-Ptc antibody to show Ptc protein expression (E), and wing discs expressing Flag:Smurf driven by *ap-gal4* were immunostained to show the expression of Ptc (red) and GFP(green), which shows the area of Smurf expression (F–F″). (G and H–H″) Wild-type control discs were immunostained with anti-Ptc antibody to show Ptc protein expression (G), and wing discs expressing Src:Flag:Smurf driven by *ap-gal4* were immunostained to show the expression of Ptc (red) and Flag (green) (H–H″).

Given the fact that the high level of *ptc* expression is directly induced by Hh but negatively regulates Hh signaling, collectively, our findings imply that a bidirectional regulation mechanism is involved in Ptc and Smurf to control and balance the signaling activity when the cells respond to external Hh molecules. Our findings also raise an intriguing issue as to how the bidirectional regulation contributes to the dynamics of Hh signaling transduction. Hh signaling dynamics has been studied previously [37], however, how Ptc (or the ratio of ligand bound to unbound Ptc) is precisely regulated in the model remains elusive. In order to better understand the dynamic basis of how Smurf-mediated Ptc turnover contributes to the reliability of the Hh signaling transduction, we developed a mathematical model below based on the current simplified Hh signaling pathway network established in this study (Figure 6A). As pointed out by Nahmad and Stathopoulos [37], the synthesis rate of Smo/Ci is regulated by the ratio [Hh_Ptc]/[Ptc] rather than [Ptc] directly since Hh-dependent gene expression depends on the ratio of liganded to unliganded Ptc.

The concentrations of the Hh, Ptc, and Hh_Ptc complex, activated Smo/Ci, and activated Smurf are denoted in the equations below by [Hh], [Ptc], [Hh_Ptc], [activated Smo/Ci], and [activated Smurf], respectively, and, for simplicity, the synthesis rate of Hh is assumed to be a constant *C_Hh_* [36],[37]. However, for convenience, let *x*_1_ = [Hh], *x*_2_ = [Ptc], *x*_3_ = [Hh_Ptc], *x*_4_ = [activated Smo/Ci], and *x*_5_ = [activated Smurf]. Thus, according to Figure 6A, the rate equations of [Hh], [Ptc], [Hh_Ptc], [activated Smo/Ci], and [activated Smurf] can be given by
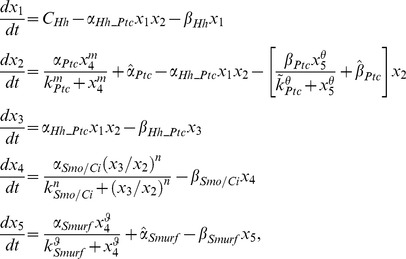
(1)


where the terms 
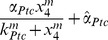
, 
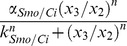
, and 
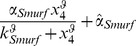
 are the Hill-type functions regarding the synthesis rates of Ptc, activated Smo/Ci, and activated Smurf, respectively, where 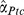 and 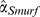 are the fundamental synthesis rates of Ptc and Smurf; 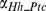 is the formation rates of Hh_Ptc complex; and the terms 
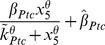
 is the Hill-type functions regarding the degradation rates of Ptc, where 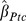 is the fundamental degradation rate of Ptc; and 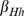, 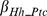, 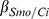, and 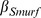 are the degradation rates of Hh, Hh_Ptc complex, activated Smo/Ci, and activated Smurf, respectively, which are assumed to be constants.

Basically, the stability analysis of Equation 1 (see Table S2) shows that the dynamics has a unique equilibrium and is globally asymptotically stable. We also noticed that although the change in the level of activated Smurf cannot change the dynamical properties of the system (i.e., no multi-equilibrium and periodic or chaotic solutions can exist), the levels of Ptc (or the ratio [Hh−Ptc]/[Ptc]) and activity of Smo/Ci sensitively depend on the change in the levels of activated Smurf. That is, the increase of activated Smurf will result in the decrease of Ptc (or the increase of the ratio [Hh−Ptc]/[Ptc]) and increase in the activity of activated Smo/Ci, and conversely. Numerical solutions of Equation 1 are shown in Figure 6B–6D, in which the effects of different activated Smurf levels on Ptc and activated Smo/Ci are considered. It appears that the theoretical and numerical analysis of Equation 1 matches our experimental data.

The above dynamic analysis reveals how the bidirectional regulation of activated Smo/Ci on Ptc, namely, activated Smo/Ci directly promotes the synthesis of Ptc through transcriptional regulation, but or indirectly promotes the degradation of Ptc through promoting the activity of Smurf. This bidirectional control appears to affect the dynamical properties of the Hh signaling pathway, and this mathematic model reveals that Ptc (or the ratio of [Hh−Ptc]/[Ptc]) not only responds to the Hh signaling but also depends more sensitively on the bidirectional regulation of activated Smo/Ci. Thus, theoretically, the bidirectional regulation occurring in Ptc and Smurf should be able to ensure the reliability of Hh signaling transduction when cells respond to an external Hh signal.

### Plasma Membrane-Tethered Smurf Enhances Its Activity to Regulate Ptc Turnover

The role of Smurf in targeting Ptc protein indicates the biological importance of its potential localization on plasma membrane. On the basis of our mathematic model, we predicted that an increase of Smurf concentration (or activity) on plasma membrane would accelerate the turnover of Ptc protein, and subsequently reduce the level of Ptc. To further test our model, we generated transgenic lines, P{*uas-Src:Flag:smurf*}, in which a membrane-tethered form of Smurf was under the control of the UAS promoter. Expression of the membrane-tethered form of Smurf *(Src:Flag:smurf)* appeared to accumulate Smurf on plasma membrane, compared to the wild type of Smurf (Figure S6A and S6B–S6B′). As shown in Figure 6E–6H″, while expression of wild-type Smurf by the *ap-gal4* driver in the dorsal region of wing discs caused a weak reduction of Ptc protein (Figure 6E and 6F–6F″), expression of the membrane-tethered form of Smurf almost abolished Ptc protein expression (Figure 6H–6H″) and increased *dpp-lacZ* expression by *MS1096-gal4* (Figure S6C, S6D–6D″, and S6E–S6E″), suggesting that the membrane anchorage of Smurf appears to increase its activity in controlling Hh signaling, and further supporting our mathematic model that Smurf plays an important role in targeting Ptc protein degradation, thereby balancing Hh signaling.

### Smurf Proteins Have a Conserved Role in Regulating Hh Signaling by Targeting Ptc

**Figure 7:**
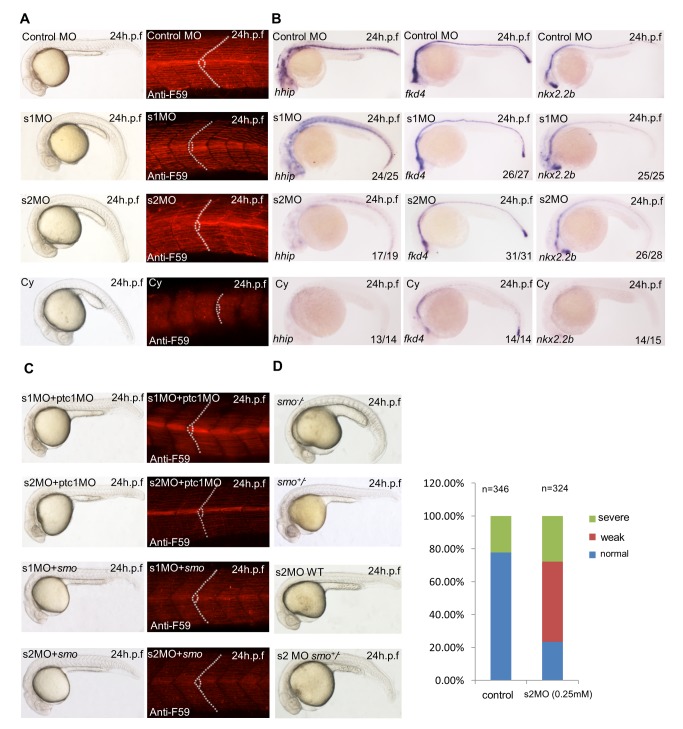
Smurfs are involved in the regulation of hedgehog signaling pathway in zebrafish. (A) *smurf1* and *smurf2* morphants mimic loss-of-Hh phenotype. Compared with control MO-injected embryos (top panels), embryos injected with 4 ng of *smurf1*-atg MO (s1MO) or of *smurf2*-atg MO (s2MO) displayed weakly curled tails, U-shaped somite, and disrupted somitic muscles of the mid-trunk region, as revealed by anti-F59 immunostaining (middle panels), which are similar to but weaker than cyclopamine-treated embryos (bottom panels). (B) Downregulation of three Hh-responsive target genes, *hhip*, *fkd4*, and *nkx2.2b* in *smurf* MOs. (C) Co-injection of *smurf* MOs (4 ng) with 4 ng *ptc1*MO (top two panels) or 100 pg *smo* mRNA (bottom two panels) can rescue the Hh-like phenotype of *smurf1* or *smurf2* MOs. (D) Injection of a low dose of s2MO (2 ng) into *smo* ^+/−^ embryos resulted in an Hh-like phenotype (bottom panel), but did not show any phenotype in the WT (*smo* ^+/+^) embryos (middle panel). Statistical data are shown in the right panel. *smo* ^−/−^ embryos showed a typical Hh phenotype (top panel), while *smo* ^+/−^ embryos were normal (middle panel).

To this end, we have identified a novel role of Smurf in regulating Hh signaling in *Drosophila*. Given that both Smurf and the Hh signal pathway are evolutionarily conserved from fly to vertebrates, we then turned our attention to test whether Smurf proteins are also involved in regulating Hh signaling in vertebrates using zebrafish embryos as an assay system. The zebrafish genome harbors two *smurf* genes, *smurf1* and *smurf2*, which encode zebrafish Smurf1 and Smurf2 proteins, respectively. As shown in an *in situ* hybridization assay, both *smurf1* and *smurf2* mRNAs were ubiquitously present in all stages during embryogenesis (Figure S7A). To investigate the biological functions of Smurfs in zebrafish embryonic development, we performed morpholino (MO)-based knockdown experiments to inactivate *smurf1* or *smurf2* in zebrafish embryos. As shown in Figure 7A, both *smurf1* and *smurf2* morphants displayed curved tails and U-shaped somite phenotypes, compared with control MO injected embryos. Injection of another splicing blocking morpholino, *smurf1* s1MO, which blocks the intron 3 splicing from the primary *smurf1* transcript, thereby causing intron 3 retention, gave similar somitic phenotypes (Figure S7B) (unpublished data). Whole-mount F59 immunofluorescence staining analysis clearly showed that somitic muscles in the mid-trunk region were disrupted in the *smurf1* or *smurf2* morphants (Figure 7A). These Hh-related defects were typically observed in the *smo* mutant or in embryos treated with cyclopamine (Figure 7A and 7D), which specifically blocks Hh signaling in zebrafish [38], indicating a potential role of Smurf proteins in regulating Hh signaling that controls the somite development (i.e., specification of distinct muscle cell types). To validate whether these phenotypes resulting from inactivated *smurfs* are attributed to disruption of Hh signaling, we first examined the expression levels of several Hh responsive target genes, such as *hhip*, *fkd4*, and *nkx2.2b* in *smurf* morphants. Consistent with the phenotypic analysis, the expression levels of these Hh responsive target genes were significantly reduced in either *smurf1* or *smurf2* morphants when compared with the control morphants (Figure 7B), suggesting that Hh signaling activity was indeed downregulated when either *smurf1* or *smurf2* was inactivated. Expression of Engrailed, a direct target of Hh signaling, was also reduced or absent in *smurf1* or *smurf2* morphants (Figure S7C). We then elevated Hh signaling in *smurf* morphants by injection of *smo* mRNA. Interestingly, we found that co-injection of *smo* mRNA appeared to suppress the phenotypes resulting from either *smurf1* or *smurf2* MOs (Figure 7C). To further confirm the role of Smurfs in regulating Hh signaling in zebrafish, we performed a dosage-modified genetic interaction between Smurfs and Hh by employing *smo* mutants. As shown in Figure 7D, both embryos injected with low doses of *smurf1* or *smurf2* MOs (2 ng/embryo) and embryos carrying heterozygous *smo* mutant exhibited no apparent phenotype during embryo development. However, *smo* heterozygous embryos showed strong Hh-related phenotypes when the embryos were injected with the same dose of *smurf1* or *smurf2* MOs.

To test whether Smurfs have a role in the degradation of Ptc1 in zebrafish, we made a zebrafish *Ptc1c* and *GFP* fusion mRNA (*Ptc1c:GFP*). As shown in Figure S4D, co-injection with either *Smurf1 or Smurf2* mRNA resulted in much weaker fluorescence, compared with *Ptc1c:GFP* mRNA injection alone, suggesting that *Smurfs* might play a conserved role in degrading Ptc protein. Similarly, knockdown of *Ptc1* could efficiently rescue the somite defects in *smurf* morphants (Figure 7C), suggesting that Smurfs negatively regulates Ptc1. Collectively, our results support the notion that zebrafish Smurf1 and Smurf2 also function as positive modulators in Hh signaling via targeting the Ptc1 protein for its degradation, thereby controlling late somite development (i.e., distinct muscle cell types) during zebrafish embryogenesis.

## Discussion

We have previously shown that Smurf functions in concert with Fu to degrade the BMP Tkv receptor in differentiating cystoblasts (CBs), thereby generating a steep BMP responsive gradient between germline stem cell (GSC) and CBs, and determining the GSC fate in *Drosophila* ovary [24]. Thus, our prior findings reveal an important *in vivo* role of Smurf E3 ligase in targeting to the membrane receptor for proper BMP signal transduction. In this study, we provide both biochemical and genetic evidence to show a novel role of Smurf in mediating Hh signal transduction by targeting to Ptc for its degradation in wing discs. Additionally, we find that, like their homologue in *Drosophila*, the zebrafish Smurf proteins are also involved in Hh signaling via targeting to the Ptc1 protein to control late somitogenesis during zebrafish embryogenesis. Thus, our study reveals a novel and evolutionarily conserved role of Smurf proteins in controlling Hh signaling.

Smurf is a member of the C2-WW-HECT E3 family of proteins in *Drosophila*. In addition to Smurf, *Drosophila* also has two other members of the C2-WW-HECT family of proteins: Suppressor of deltex (Su(dx)) and Nedd4 protein. Interestingly, we found that all three of these E3 ligases could individually form a complex with the Ptc protein through the C-tail of Ptc (Figures S2H, S2I, and S8A). However, we observed that Smurf is the only member of the C2-WW-HECT family in *Drosophila* that plays a positive role in the regulation of Hh signaling through targeting Ptc in the wing disc system, since alteration of Nedd4 expression did not affect Hh signaling activity and the Su(dx) has a negative role in regulating the Hh signaling pathway in the wing disc (Figure S8B–S8K). Notably, we also found that zebrafish Nedd4 has no role in Hh signaling during early embryonic development in zebrafish (Figure S9). Thus, our findings suggest a specific biological role for Smurf in targeting Ptc to balance Hh signaling in the tested systems in this study.

Morphogen-mediated signaling gradients have been proposed to regulate differential gene expression in a concentration-dependent manner [39],[40]. However, the fundamental question of how signal-receiving cells perceive and precisely interpret the environmental cues provided by morphogens has been not well-understood [39],[41]. The Hh signaling pathway has an unusual signal reception system including the receptor Ptc and the signal transducer Smo. While the activation of Smo and its downstream events have been extensively studied, how the Hh receptor Ptc is regulated and whether activation of Smo also contributes to regulating the expression and/or activity of the receptor Ptc remain unknown. Given that Ptc has a negative role in the regulation of Hh signaling and that *ptc* itself is a responsive target of Hh signaling, the expression and/or activity of Ptc must be tightly controlled to ensure proper Hh signal transduction. In *Drosophila* wing discs, previous studies have proposed that Hh ligand binding and self-induced degradation of Ptc might contribute to the Ptc internalization and the regulation of Ptc expression [13],[30]. In this study, we identify Smurf as a Ptc-interacting partner, and provide evidence that Smurf functions as a negative regulator to control Ptc ubiquitination and degradation. Importantly, we observe that Smo activation promotes the E3 ligase activity of Smurf to ubiquitinate Ptc protein in both wing discs and S2 cell cultures. In particular, we find that Smurf activity largely depends on the activation of Smo to regulate Ptc turnover. Previous studies have shown that plasma membrane accumulation of Smo is essential for Hh signaling transduction [42]. In this study, we show that Smurf was able to form a complex with Smo to regulate Ptc ubiquitination in a manner that depends on the specific Ptc-K1261 site. Thus, in addition to establishing functions for Hh signal transduction by controlling Ci processing, our findings identify a novel role for the Smo protein in targeting the Ptc receptor on plasma membrane for Ptc degradation.

Previous studies have proposed an elegant model by which the ratio of ligand bound to unbound Ptc protein is important in determining Hh signaling activity in *Drosophila* wing discs [36]. However, the molecular mechanism underlying the regulation of the ratio of ligand bound to unbound Ptc remains unknown. Given that the *ptc* itself is a transcriptional target of the Hh signal, and that the excessive unbound Ptc induced by Hh signaling appears to potentially increase fluctuating levels of cellular Hh signaling, the question becomes how excessive Ptc is restricted within a safe range to control the appropriate ratio of ligand bound to unbound Ptc. Notably, our findings reveal that Smurf has a preference for degrading unbound Ptc (Figure 4C–4J); thus, integration of Smurf-mediated Ptc degradation with Hh signaling permits us to propose a bidirectional regulatory model that maintains the robustness of cellular Hh responsive activity through controlling a precise ratio of ligand bound to unbound Ptc. In this model, in the presence of Hh molecules, Ptc is highly expressed in signal-receiving cells in response to Hh signaling activation, but negatively regulates Hh signaling through a feedback mechanism. Meanwhile, Smurf also responds to activation of Smo and forms a complex with Smo, thus accumulating on plasma membrane to target Ptc for degrading the excessive unbound Ptc protein, thereby controlling the ratio of bound to unbound Ptc protein and subsequently sustaining the precise level of Hh signaling that ensures distinct target gene activations during development (Figure 6A). The mathematic modeling analysis reveals that Ptc not only responds to the Hh signaling but also depends more sensitively on the bidirectional regulation of activated Smo/Ci, further supporting the importance of the role of Smurf in controlling the reliability of Hh signal transduction.

Hh signaling is the fundamental signaling pathway, and is critical for development and stem cell function in adult tissues [2],[3]. Aberrant regulation of Hh signaling has been implicated in many human diseases, particularly cancers. Clinical examples include basal cell carcinoma (BCC), medulloblastoma, and other human tumors that are usually associated with mutations of Hh signaling components including PTC1 and SMO [43]–[45]. Interestingly, a very recent study has provided a link between the function of Smurf2 and tumorigenesis, since loss of Smurf2 leads to various types of tumors in old age [46]. Given that Smurf proteins are evolutionarily conserved from *Drosophila* to vertebrates and that the molecular basis of Ptc protein degradation is likely conserved in evolution [13],[47], a target of future investigation would be, therefore, whether Smurf is involved in regulating Hh signaling-related tumorigenesis.

## Materials and Methods

### Fly Stocks

Fly stocks used in this study were maintained under standard culture conditions. The *w^1118^* strain was used as the host for all P element-mediated transformations. *smurf^15c^*, *MS1096-gal4*, *ap-gal4*, *act>CD2>gal4*, *UAS-GFP*, *dpp–lacZ (dppZ)*, *ptc–lacZ(ptcZ)*, UAS-Hh, UAS-Smo^SD12^, UAS-Smo^SD123^, and UAS-HA:Ci^103^ have been described previously [11],[20],[48],[49]. Su(dx) RNAi, (#N4244R-1), and Nedd4 RNAi (#V13121) were obtained from NIG and VDRC. Fly Strains including P{*uast-Flag-smurf*}, P{*uast-Flag-smurf^C1029A^*}, P{*uast-SRC-Flag-smurf*}, P{*uast-Flag-su(dx)*}, P{*uast-Flag-nedd4*}, P{*hs-ptc:gfp*}, P{*uast-ptc*}, P{*uast-ptc^K1261R^*}, and P{*uasp-shmiR-smurf*} [35] were generated in this study.

### Mosaic Clonal Assay

The flies *y w P{hsFLP}122/y w P{hsFLP}122 or Y; P{FRT(w[hs])}G13 2xP{Ubi-GFP.nls}/P{FRT(w[hs])}G13 Smurf^15c^; dpp-lacZ* were used for generating *smurf* mosaic clones in wing discs. Larvae were heat shocked 2–3 days after birth at 37°C for 30 min to induce flippase activity, and then cultured at 25°C for another 2–3 days for wing disc dissection.

### Immunohistochemistry for *Drosophila* Wing Discs

Standard protocols for immunofluorescence staining of imaginal discs were used [50]. The following antibodies were used in this study, including rabbit anti-GFP (1∶5,000, Invitrogen); mouse anti-β Gal (1∶1,000 Promega); rabbit anti-β Gal (1∶2,000, Cappel); mouse anti-Flag (1∶500, Sigma); rabbit anti-Flag (1∶500, Sigma); rat anti-Ci (1∶200, DSHB); mouse anti-Ptc (1∶200, DSHB); and mouse anti-Smo (1∶500, DSHB). The following secondary antibodies were used at a 1∶2,000 dilution: goat anti-mouse Alexa555, goat anti-rabbit Alexa488, and goat anti-rat Alexa647 (Molecular Probes). CHX treatments of wing discs were performed in M3 medium containing 2% fetal bovine serum (Hyclone), 2.5% fly extract, and 0.5 mg/ml insulin (Sigma) for 2 h at 25°C.

### GST Fusion Protein Pull-Down Assay

GST and GST:Flag:Ptc^CTD^ fusion proteins were expressed in *Escherichia coli*, and purified with glutathione agarose beads (GE Healthcare) by the batch purification method. His:Smurf protein was expressed in *E. coli*, and purified with Ni Sepharose (GE Healthcare), and GST fusion protein loaded beads were incubated with 200 ng of purified His:Smurf in GST pull down buffer (50 mM Tris-Cl [pH 8.0], 200 mM NaCl, 1 mM EDTA, 1% NP-40, 2% BSA, 10 mM DTT, 1 mM PMSF) at 4°C for 1 h. The beads were washed three times with lysis buffer. Western blot analysis was then performed to detect the direct interaction between Smurf and Ptc^CTD^.

### Cell Culture, Immunoprecipitation, and Western Blot Analysis

S2 cells were cultured in Schneider's *Drosophila* medium (Sigma) at 27°C. Transfection was performed using the calcium phosphate transfection method according to our previous method [51]. In some experiments, transfected S2 cells were treated with MG132 (50 µM) or NH_4_Cl (50 mM) for 4 h before harvesting when necessary. Cells were lysed in lysis buffer (150 mM NaCl, 50 mM Tris-HCl [at pH 7.4], 10% glycerol, 1% Triton X-100) with a protease-inhibitor cocktail (Invitrogen), before binding with affinity gel; the concentration of NaCl in cell lysate was adjusted to 500 mM. Anti-Flag M2 affinity gel (Sigma), anti-Myc affinity gel (Abmart), and protein A/G gel were used for indicated immunoprecipitation experiments. After affinity pull-down, gels were extensively washed in lysis buffer contain 500 mM NaCl for 15 min each time, and for a total of three times. Western blot analysis of full-length Ptc protein was carried out by SDS-PAGE electrophoresis without boiling; the other proteins were analyzed by using standard protocol. The following antibodies were used for Western blotting: rabbit and mouse anti-Myc (1∶3,000, MBL); rabbit anti-HA (1∶3,000, MBL); rabbit and mouse anti-Flag (1∶3,000, Sigma); mouse anti-Ptc (1∶1,000, DSHB); mouse anti-Smurf (1∶10,000) [24]; and rabbit anti-α-tubulin (1∶5,000, Abcam).

### *In Vivo* Ubiquitination Assay

*In vivo* ubiquitination assays were performed according to our previous study [31]. Briefly, S2 cells were transfected with indicated DNA constructs. At 48 h post-transfection, cells were treated with MG132 (at a final concentration of 50 µM) and/or NH_4_Cl (at a final concentration of 50 mM) for 4 h. Cells were lysed in lysis buffer (50 mM Tris-HCl [at pH 7.4], 150 mM NaCl, 10% glycerol, 1% Triton X-100, 0.1% SDS, and 10 mM NEM) with a protease-inhibitor cocktail (Invitrogen). Supernatant was collected by centrifugation at 13,000 rpm for 20 min at 4°C. Before immunoprecipitation with anti-Flag or anti-Myc beads, NaCl concentration in the lysates was adjusted to 500 mM. After pull-down for 4 h, the beads were then extensively washed with lysis buffer containing 0.1% SDS and 500 mM NaCl three times for a total 1 h. Samples were then subjected to Western blot analysis.

In addition to the method described here, we also used two other methods to measure Ptc ubiquitination levels (described in Text S1); we noted that all three methods generated consistent results.

## Supporting Information

Figure S1 **Smurf regulates Ptc ubiquitination through its C-tail.** (A) The Flag tagged Ptc^CTD^ (1101–1286 a.a) or Ptc^CTD^ carrying PPAY mutations (PPAY-AAAA) was sub-cloned into the pGEX4T-1 vector, and the full length of *Drosophila* Smurf was cloned into the pET28a vector. The bacterially expressed proteins were then purified with the glutathione sepharose 4 fast flow and the ni sepharose systems, respectively. GST pull down assays was performed to detect the direct protein interaction between Smurf and Ptc^CTD^ or Ptc^CTD(PPXY-AAAA)^. (B) S2 cells were transfected with combinations of DNA constructs as indicated. After 48 h transfection, lysates from transfected S2 cells were subjected to Western blot assays and showing expression levels of Ptc and its mutants as indicated. (C and D) S2 cells were transfected with combinations of DNA constructs as indicated. After 48 h transfection, S2 cells were treated with MG132 (50 µM final concentration) (C) or with NH_4_Cl (50 mM final concentration) for 4 h (D). Harvested S2 cells were treated with denaturing buffer at 100°C for 10 min and then immunoprecipitated with mouse anti-Myc affinity gel. Western blotting was performed to analyze the presence of indicated proteins and levels of ubiquitination of Ptc. (E and F) S2 cells were transfected with combinations of DNA constructs as indicated. After 48 h transfection, S2 cells were treated with MG132 (50 µM final concentration) (E) or with NH_4_Cl (50 mM final concentration) for 4 h (F) for 4 h. Harvested S2 cells were treated with denaturing buffer at 100°C for 10 min and then immunoprecipitated with rabbit anti-HA antibody and protein A/G Sepharose beads. Western blotting was then performed to analyze the presence of indicated proteins and levels of ubiquitination of Ptc.
(PDF)

Figure S2 **Smurf regulates Ptc ubiquitination through its C-tail.** (A) S2 cells were transfected with combinations of DNA constructs as indicated. After 48 h transfection, S2 cells were treated with MG132 (50 µM final concentration) and NH_4_Cl (50 mM final concentration) for 4 h. Cell lysates were then immunoprecipitated with mouse anti-Myc affinity gel. Western blotting was used to analyze the presence of indicated proteins and levels of ubiquitination of Ptc^CTD^. (B and C) S2 cells were transfected with combinations of DNA constructs as indicated. After 48 h transfection, S2 cells were treated with MG132 (50 µM final concentration) and NH_4_Cl (50 mM final concentration) for 4 h. Harvested S2 cells were treated with denaturing buffer for 10 min and then immunoprecipitated with mouse anti-Myc affinity gel (B) or mouse anti-Flag affinity gel (C). Western blotting was performed to analyze the presence of indicated proteins and levels of ubiquitination of Ptc. (D) The panel shows the same Figure as Figure 2H, but a longer exposure time was used. (E) S2 cells were transfected with combinations of DNA constructs as indicated. After 48 h transfection, S2 cells were treated with MG132 (50 µM final concentration) and NH_4_Cl (50 mM final concentration) for 4 h. Harvested S2 cells were treated with denaturing buffer for 10 min and then immunoprecipitated with rabbit anti-HA antibody and protein A/G Sepharose beads. Western blotting was performed to analyze the presence of indicated proteins and levels of ubiquitination of Ptc. (F) S2 cells were transfected with combinations of DNA constructs as indicated. After 48 h transfection, lysates from transfected S2 cells were immunoprecipitated with anti-Flag M2 affinity gel. Western blots were performed to analyze the presence of Flag-tagged or Myc-tagged proteins. (G) An *in vitro* ubiquitination assay was performed according to the method in Text S1. After reaction, proteins were immuno-purified with anti-Ub antibody and Protein A/G Sepharose beads, and anti-Flag antibody was used to detect Ptc^CTD^ ubiquitination in Western blot assays. (H and I) S2 cells were transfected with combinations of DNA constructs as indicated. After 48 h transfection, lysates from transfected S2 cells were immunoprecipitated with anti-Flag M2 affinity gel. Western blots were performed to analyze the presence of Flag- or Myc-tagged proteins. These results show that, like Smurf, Nedd4 and Su(dx) could physically interact with Ptc through their C-tails.
(PDF)

Figure S3 **Smurf regulates Hh signaling by controlling Ptc turnover.** (A–A″) Wing discs expressing Flag:Smurf driven by *ap-gal4* were immunostained to show the expression of *dppZ* (red) and Flag (green). (B–B″) Wing discs expressing Flag:Smurf^C1029A^ driven by *ap-gal4* were immunostained to show the expression of *dppZ* (red) and Flag (green). (C–C″) Wing discs expressing shmiR *smurf* and uas-GFP by *ap-gal4* were immunostained to show the expression of *dppZ* (red) and GFP (green). (D) Wild-type control discs were immunostained with anti-Ptc antibody to show Ptc protein expression at the same time as (E–J). (E–J) Wing discs with indicated genotypes were dissected into complete M3 medium and treated with control solvent DMSO (E–G) or CHX (H–J) for 2 h, and immunostaining was then performed to show Ptc protein levels. (K) Wing discs with indicated genotypes were dissected into complete M3 medium and treated with control solvent or CHX for 2 h; 100 discs for each lane were collected. Western blots were performed to show the level of Ptc protein; α-tubulin was used as loading control.
(PDF)

Figure S4 **Smurf interacts with Smo but has no apparent role in regulating Smo stability.** (A) Wing discs expressing constitutively activated form of Ci with the HA tag (HA:Ci^103^) driven by *MS1096-gal4* were stained with anti-Ptc antibody to show the expression of Ptc in discs. (B) Wing discs expressing both HA:Ci^103^ and Flag:Smurf driven by *MS1096-gal4* were immunostained to show the expression of Ptc. (C) Wing discs expressing constitutive form of Ci (Ci^103^) and Flag:Smurf^C1029A^ driven by *MS1096-gal4* were immunostained to show the expression of Ptc. (D) Wing discs expressing the indicated transgene driven by *MS1096-gal4* were dissected and were used for Western blot assay to show levels of Ptc protein in each genotypes. (E) S2 cells were transfected with combinations of DNA constructs as indicated. Smo^ΔC^ represents 1–555 a.a of Smo and Smo^ΔN^ represents 556–1,036 a.a of Smo. After 48 h transfection, lysates from transfected S2 cells were immunoprecipitated with anti-Flag M2 affinity gel. Western blots were performed to analyze the presence of Flag-tagged or Myc-tagged proteins. These results revealed that Smo was associated with Smurf through its C-tail. (F) S2 cells were first treated with indicated dsRNA for 48 h, and then transfected with combinations of DNA constructs as indicated. After 48 h transfection, S2 cells were treated with MG132 (50 µM final concentration) and NH_4_Cl (50 mM final concentration) for 4 h. Cell lysates were immunoprecipitated with mouse anti-Myc affinity gel. Western blotting was performed to analyze the presence of indicated proteins and levels of ubiquitination of Smo. These results suggested that Smurf did not affect the ubiquitination of Smo. (G) S2 cells were treated with indicated dsRNA for 72 h, and then transfected with Smo:Myc construct, Western blot was performed to measure levels of Smo protein. (H–I′″) Flies with the indicated genotype were treated by heat-shock to induce mosaic cell clones using Flip-out method, wing discs carrying clones expression of *smurf* knockdown transgene were immunostained to show the expression of GFP (green), Smo (red), and Ci (purple). Cell clones with knockdown of *smurf* were marked by the GFP+. (I, I′, I″, and I′″) show the enlarged image of the part boxed with white lines in (H, H′, H″, and H′″), respectively.
(PDF)

Figure S5 **Smurf regulates Ptc ubiquitination through specific sites in its C-tail.** (A) S2 cells were transfected with combinations of DNA constructs as indicated, after 48 h transfection, S2 cells were treated with MG132 (50 µM) and NH_4_Cl (50 mM) for 4 h. Lysates were then immunoprecipitated with mouse anti-Myc affinity gel. Western blotting was used to analyze the presence of indicated proteins and levels of ubiquitination of Ptc and its mutants. (B) S2 cells were transfected with indicated constructs for 48 h, cell lysates were subjected to Western blot by using the native polyacrylamide gel method to detect the trimer of Ptc^CTD^.
(PDF)

Figure S6 **Membrane-tethered Smurf enhances its activity to regulate Hh signaling.** (A–B′) Wing discs expressing Flag:Smurf (A) or Src:Flag-Smurf (B–B′) driven by *ptc-gal4* were immunostained to show the localization of Smurf. (C) WT wing discs carrying *dpp-LacZ* (*dppZ*) reporter were immunostained with anti-β Gal antibody to show the expression of *dppZ* (green). (D–D″) Wing discs expressing Src:Flag:Smurf driven by *MS1096-gal4* were immunostained to show the expression of *dppZ* (green) and Flag (red). (E–E″) Wing discs from early 3rd instar larva expressing Src:Flag:Smurf driven by *MS1096-gal4* were immunostained to show the expression of *dppZ* (green) and Ptc (blue).
(PDF)

Figure S7 **Expression patterns of** ***smurf1*** **and** ***smurf2*** **during zebrafish embryogenesis. Validation of** ***smurf1*** **splicing MO, and degradation of Ptc:GFP fusion protein by Smurfs.** (A) Both *smurf1* and *smurf2* mRNA are ubiquitously expressed in all stages of zebrafish embryonic development examined. (B) The specific effect of *smurf1* splicing MO, which leads to retention of intron 3. (C) Downregulation of Hh-responsive target genes *engrailed* in *smurf* MOs. (D) Embryos injected with 100 pg ptc1-C-GFP mRNA show obvious GFP fluorescence at 20 hours post-fertilization (hpf) and 24 hpf. After co-injection of smurf mRNAs with ptc1-C-GFP mRNA, GFP expression was reduced. While embryos injected with 100 pg ptc1-N-GFP mRNA expressed weak GFP at 20 hpf and 24 hpf, there was no obvious change in embryos co-injected with smurf mRNAs and ptc1-N-GFP mRNA.
(PDF)

Figure S8 **Nedd4 and Su(dx) interact with Ptc through the C-tail of Ptc, but have different role from Smurf in the regulation of Hh signaling.** (A) Yeast two-hybrid was performed to show that the PPAY motif in Ptc^CTD^ is important for Ptc-Smurf direct interaction, and to show that Ptc directly interacts with Nedd4 and Su(dx). The AH109 yeast strain were transformed with the indicated plasmids and plated at permissive (−Leu/−Trp) and restrictive (−Ade/−His/−Leu/−Trp) medium. Full-length *smurf* and Ptc^CTD^ were cloned into the bait vector, pGBKT7, and pACT2 is the prey vector. (B–B′″) Control wing discs from *MS1096-gal4* were immunostained to show the expression of Ptc (red), *dppZ* (green), and Ci (blue). (C) Control wing discs from *MS1096-gal4* were immunostained to show the expression of *ptcZ* (green). (D–D′″) Wing discs expressing *su(dx)* knockdown transgene driven by *MS1096-gal4* were immunostained to show the expression of Ptc (red), *dppZ* (green), and Ci (blue). (E) Wing discs expressing *su(dx)* knockdown transgene driven by *MS1096-gal4* were immunostained to show the expression of *ptcZ* (green). (F–F′″) Wing discs expressing Flag:Su(dx) driven by *MS1096-gal4* were immunostained to show the expression of Ptc (red), *dppZ* (green), and Ci (blue). (G) Wing discs expressing Flag:Su(dx) driven by *MS1096-gal4* were immunostained to show the expression of *ptcZ* (green). (H–H′″) Wing discs expressing *nedd4* knockdown transgene driven by *MS1096-gal4* were immunostained to show the expression of Ptc (red), *dppZ* (green), and Ci (blue). (I) Wing discs expressing *nedd4* knockdown transgene driven by *MS1096-gal4* were immunostained to show the expression of *ptcZ* (green). (J–J′″) Wing discs expressing Flag:Nedd4 driven by *MS1096-gal4* were immunostained to show the expression of Ptc (red), *dppZ* (green), and Ci (blue). (K) Wing discs expressing Flag:Nedd4 driven by *MS1096-gal4* were immunostained to show the expression of *ptcZ* (green).
(PDF)

Figure S9 **Zebrafish Nedd4 has no apparent role in regulating Hh signaling during embryonic development.** (A) 1 ng *nedd4* MO or control MO with equal dose of *p53* MO was injected into the zebrafish one-cell stage embryos. The *nedd4* morphants showed normal embryonic development, when compared with the control. (B) The mRNA of zebrafish *nedd4* fused with a GFP tag was co-injected with 1 ng *nedd4* MO or control MO and equal dose of *p53* MO into the zebrafish one-cell stage embryos, *nedd4* MO efficiently decreased GFP signal intensity. (C) 1 ng *nedd4* MO or control MO with equal dose of *p53* MO was injected into the zebrafish one-cell stage embryos. *nedd4* morphants were immunostained with anti-En antibody to detect expression levels of Hh target gene En (red). (D) 1 ng *nedd4* MO or control MO with equal dose of *p53* MO was injected into the zebrafish one-cell stage embryos. *In situ* hybridization was performed for *nedd4* morphants to detect expression levels of Hh target genes *hhip*, *fkd4*, and *nkx2.2b*.
(PDF)

Table S1 **Potential Smurf-interacting partners identified in a yeast two-hybrid screen.**
(PDF)

Table S2 **Parameters in** **Equation 1** **.**
(PDF)

Text S1 **Supplemental materials and methods.**
(DOCX)

## Abbreviations

GFP: green fluorescent protein
MO: morpholino
