# Supplementary figures and images for: Activation of Smurf E3 Ligase Promoted by Smoothened Regulates Hedgehog Signaling through Targeting Patched Turnover
Source: PLoS Biol. 2013 Nov 26;11(11):e1001721. doi: 10.1371/journal.pbio.1001721 (PMC3841102; doi:10.1371/journal.pbio.1001721)

**A**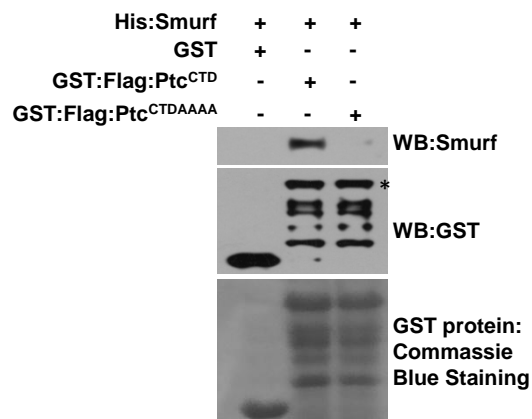**B**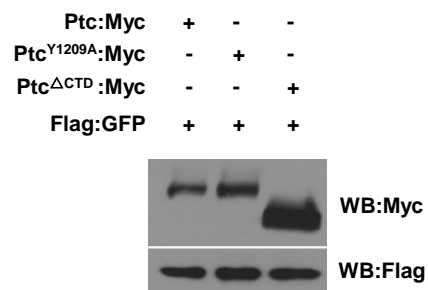**C**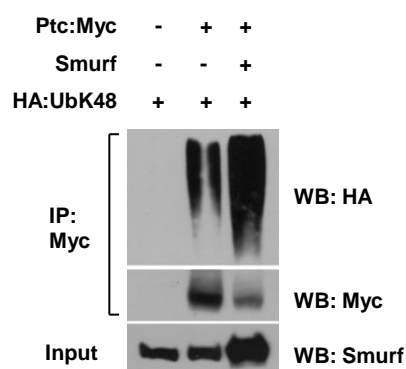**D**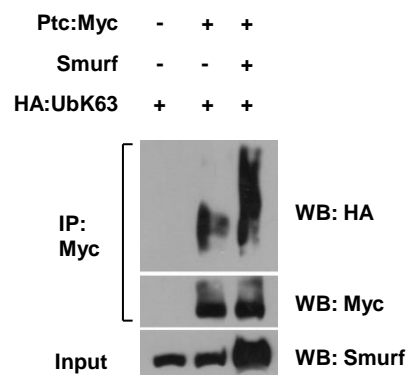**E**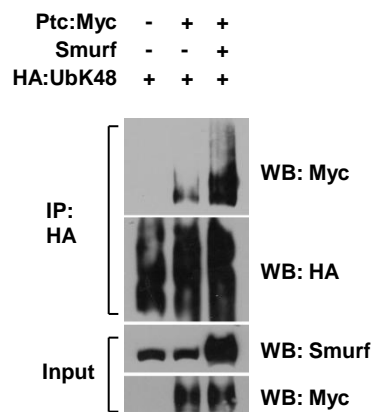**F**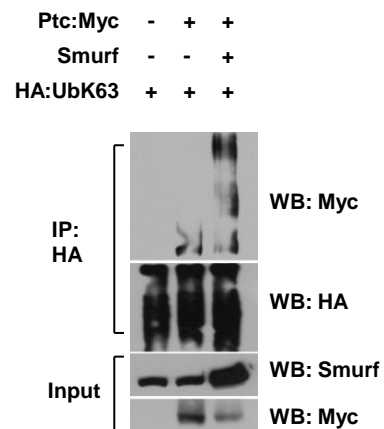

Supplement: Figure S1 — Smurf regulates Ptc ubiquitination through its C-tail. (A) The Flag tagged PtcCTD (1101–1286 a.a) or PtcCTD carrying PPAY mutations (PPAY-AAAA) was sub-cloned into the pGEX4T-1 vector, and the full length of Drosophila Smurf was cloned into the pET28a vector. The bacterially expressed proteins were then purified with the glutathione sepharose 4 fast flow and the ni sepharose systems, respectively. GST pull down assays was performed to detect the direct protein interaction between Smurf and PtcCTD or PtcCTD(PPXY-AAAA). (B) S2 cells were transfected with combinations of DNA constructs as indicated. After 48 h transfection, lysates from transfected S2 cells were subjected to Western blot assays and showing expression levels of Ptc and its mutants as indicated. (C and D) S2 cells were transfected with combinations of DNA constructs as indicated. After 48 h transfection, S2 cells were treated with MG132 (50 µM final concentration) (C) or with NH4Cl (50 mM final concentration) for 4 h (D). Harvested S2 cells were treated with denaturing buffer at 100°C for 10 min and then immunoprecipitated with mouse anti-Myc affinity gel. Western blotting was performed to analyze the presence of indicated proteins and levels of ubiquitination of Ptc. (E and F) S2 cells were transfected with combinations of DNA constructs as indicated. After 48 h transfection, S2 cells were treated with MG132 (50 µM final concentration) (E) or with NH4Cl (50 mM final concentration) for 4 h (F) for 4 h. Harvested S2 cells were treated with denaturing buffer at 100°C for 10 min and then immunoprecipitated with rabbit anti-HA antibody and protein A/G Sepharose beads. Western blotting was then performed to analyze the presence of indicated proteins and levels of ubiquitination of Ptc. (PDF) [file pbio.1001721.s001.pdf]

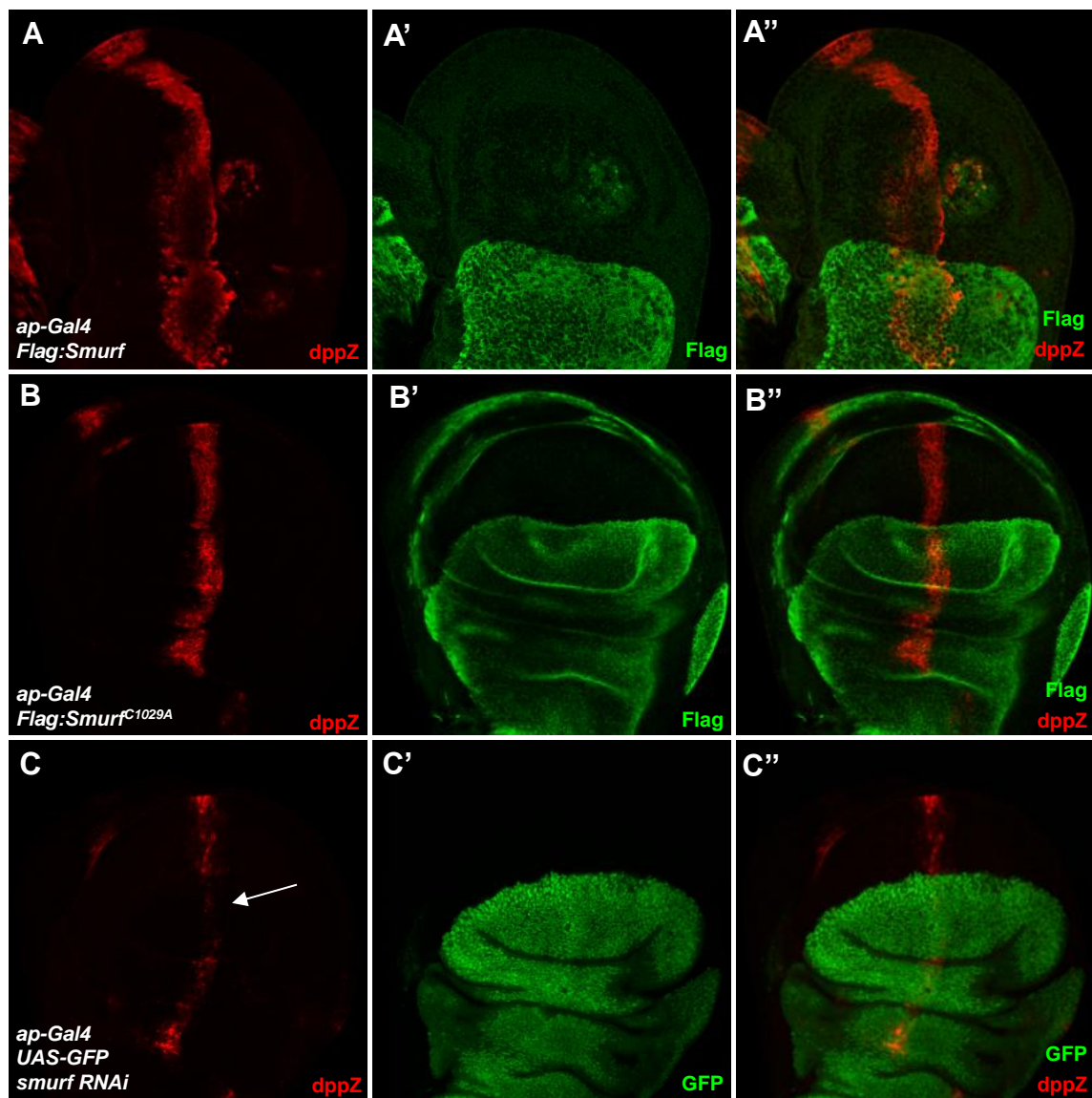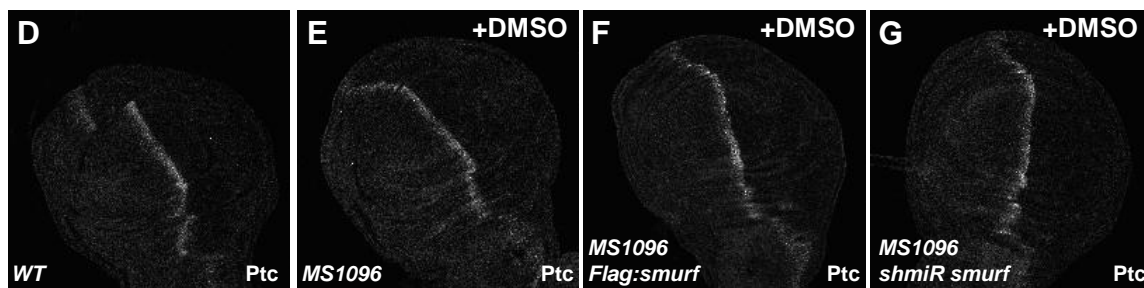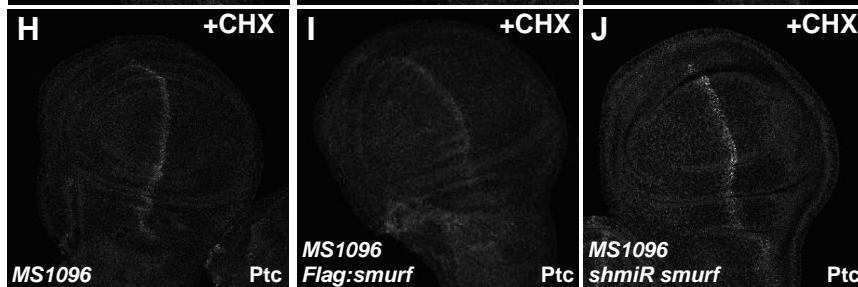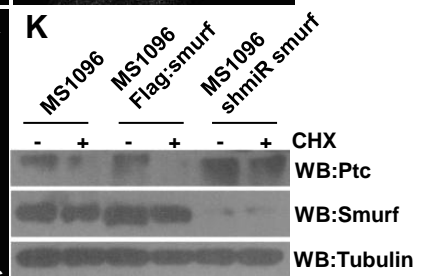

Supplement: Figure S3 — Smurf regulates Hh signaling by controlling Ptc turnover. (A–A″) Wing discs expressing Flag:Smurf driven by ap-gal4 were immunostained to show the expression of dppZ (red) and Flag (green). (B–B″) Wing discs expressing Flag:SmurfC1029A driven by ap-gal4 were immunostained to show the expression of dppZ (red) and Flag (green). (C–C″) Wing discs expressing shmiR smurf and uas-GFP by ap-gal4 were immunostained to show the expression of dppZ (red) and GFP (green). (D) Wild-type control discs were immunostained with anti-Ptc antibody to show Ptc protein expression at the same time as (E–J). (E–J) Wing discs with indicated genotypes were dissected into complete M3 medium and treated with control solvent DMSO (E–G) or CHX (H–J) for 2 h, and immunostaining was then performed to show Ptc protein levels. (K) Wing discs with indicated genotypes were dissected into complete M3 medium and treated with control solvent or CHX for 2 h; 100 discs for each lane were collected. Western blots were performed to show the level of Ptc protein; α-tubulin was used as loading control. (PDF) [file pbio.1001721.s003.pdf]

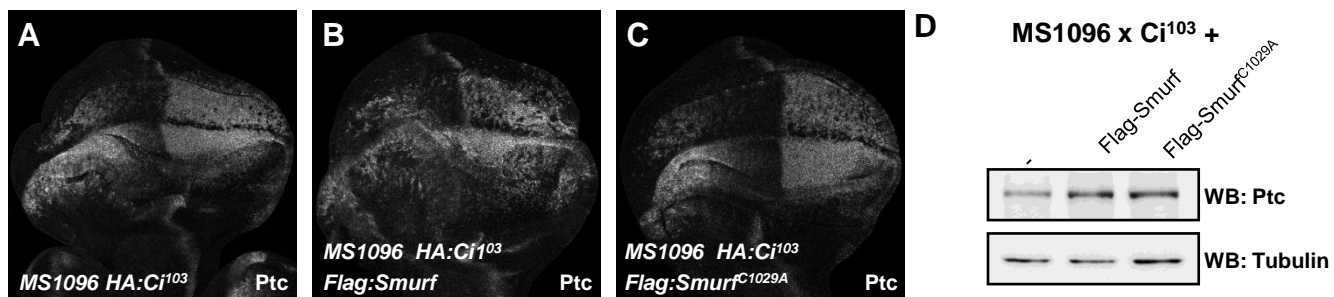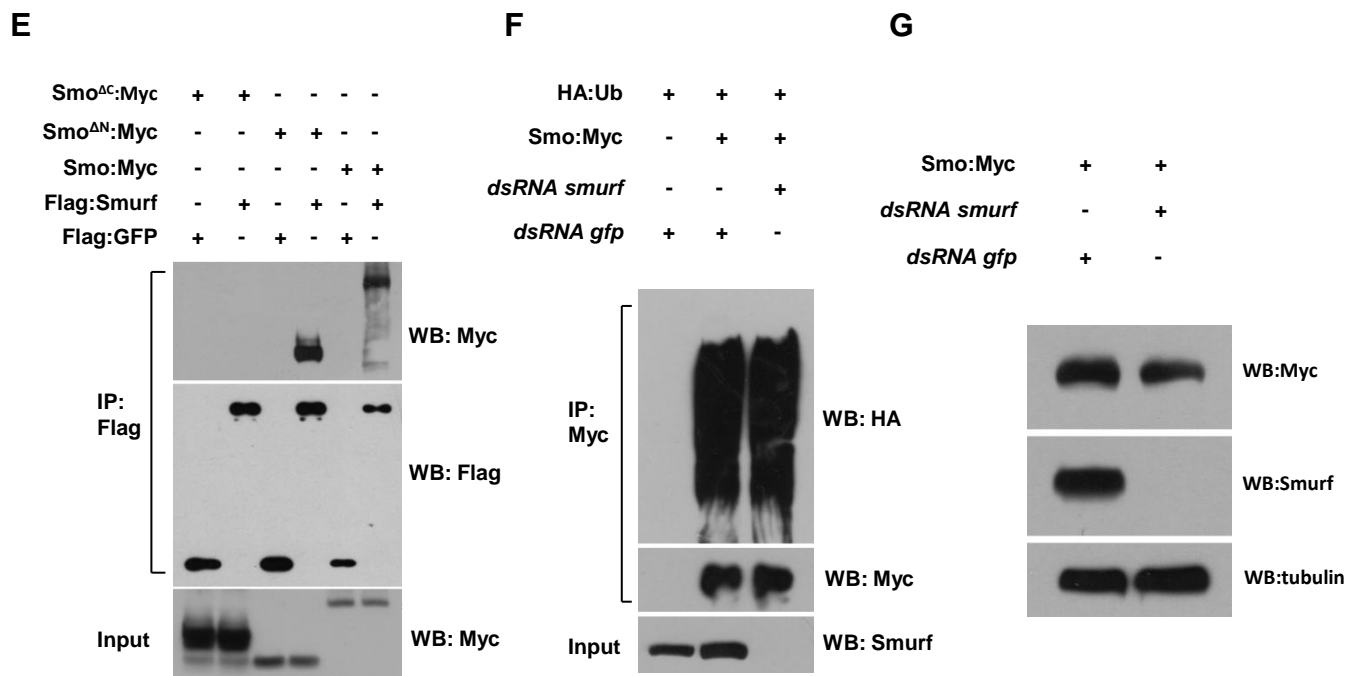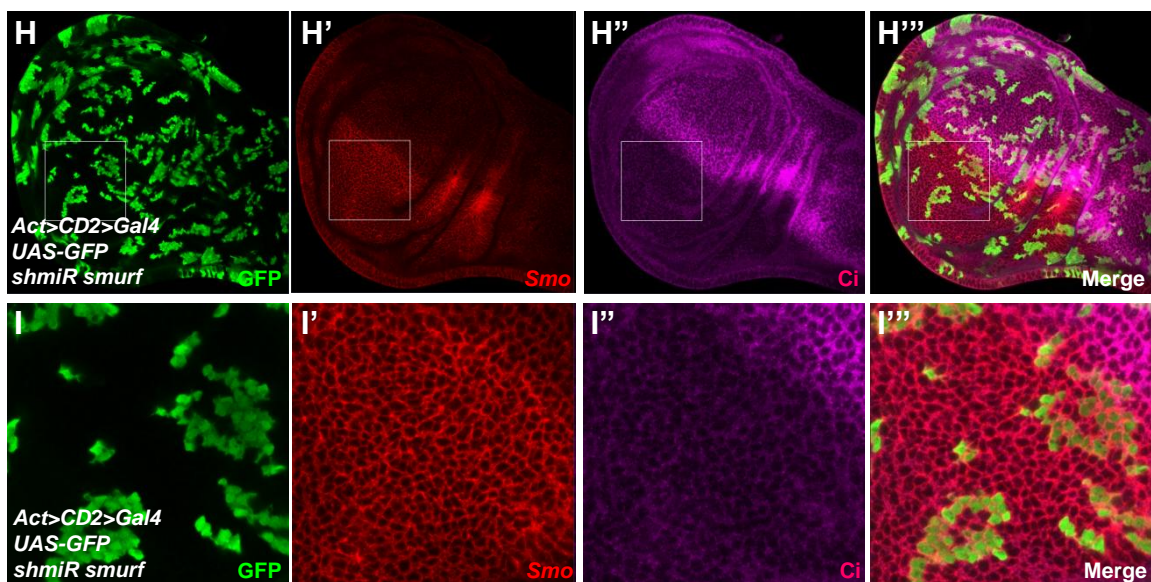

Supplement: Figure S4 — Smurf interacts with Smo but has no apparent role in regulating Smo stability. (A) Wing discs expressing constitutively activated form of Ci with the HA tag (HA:Ci103) driven by MS1096-gal4 were stained with anti-Ptc antibody to show the expression of Ptc in discs. (B) Wing discs expressing both HA:Ci103 and Flag:Smurf driven by MS1096-gal4 were immunostained to show the expression of Ptc. (C) Wing discs expressing constitutive form of Ci (Ci103) and Flag:SmurfC1029A driven by MS1096-gal4 were immunostained to show the expression of Ptc. (D) Wing discs expressing the indicated transgene driven by MS1096-gal4 were dissected and were used for Western blot assay to show levels of Ptc protein in each genotypes. (E) S2 cells were transfected with combinations of DNA constructs as indicated. SmoΔC represents 1–555 a.a of Smo and SmoΔN represents 556–1,036 a.a of Smo. After 48 h transfection, lysates from transfected S2 cells were immunoprecipitated with anti-Flag M2 affinity gel. Western blots were performed to analyze the presence of Flag-tagged or Myc-tagged proteins. These results revealed that Smo was associated with Smurf through its C-tail. (F) S2 cells were first treated with indicated dsRNA for 48 h, and then transfected with combinations of DNA constructs as indicated. After 48 h transfection, S2 cells were treated with MG132 (50 µM final concentration) and NH4Cl (50 mM final concentration) for 4 h. Cell lysates were immunoprecipitated with mouse anti-Myc affinity gel. Western blotting was performed to analyze the presence of indicated proteins and levels of ubiquitination of Smo. These results suggested that Smurf did not affect the ubiquitination of Smo. (G) S2 cells were treated with indicated dsRNA for 72 h, and then transfected with Smo:Myc construct, Western blot was performed to measure levels of Smo protein. (H–I′″) Flies with the indicated genotype were treated by heat-shock to induce mosaic cell clones using Flip-out method, wing discs carrying clones e [file pbio.1001721.s004.pdf]

**A**

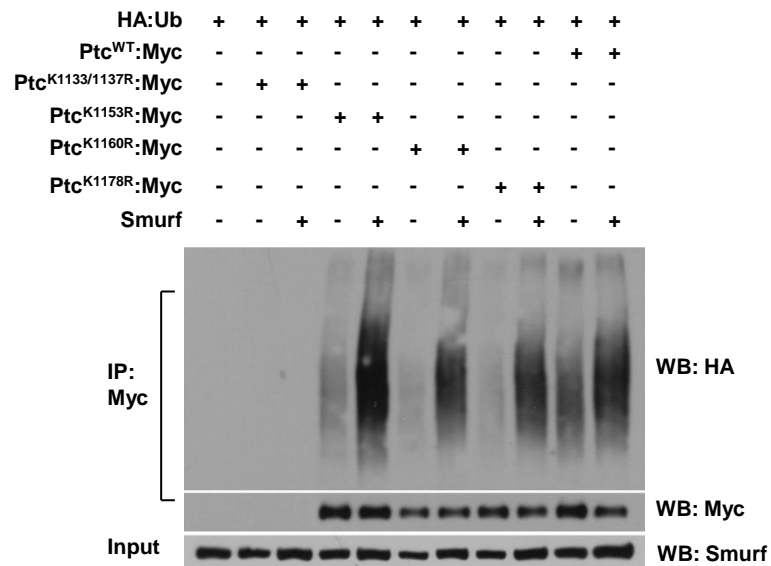

**B**

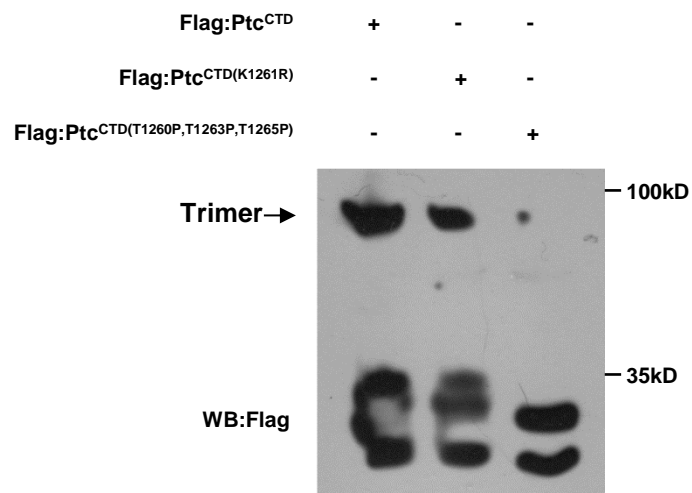

Supplement: Figure S5 — Smurf regulates Ptc ubiquitination through specific sites in its C-tail. (A) S2 cells were transfected with combinations of DNA constructs as indicated, after 48 h transfection, S2 cells were treated with MG132 (50 µM) and NH4Cl (50 mM) for 4 h. Lysates were then immunoprecipitated with mouse anti-Myc affinity gel. Western blotting was used to analyze the presence of indicated proteins and levels of ubiquitination of Ptc and its mutants. (B) S2 cells were transfected with indicated constructs for 48 h, cell lysates were subjected to Western blot by using the native polyacrylamide gel method to detect the trimer of PtcCTD. (PDF) [file pbio.1001721.s005.pdf]

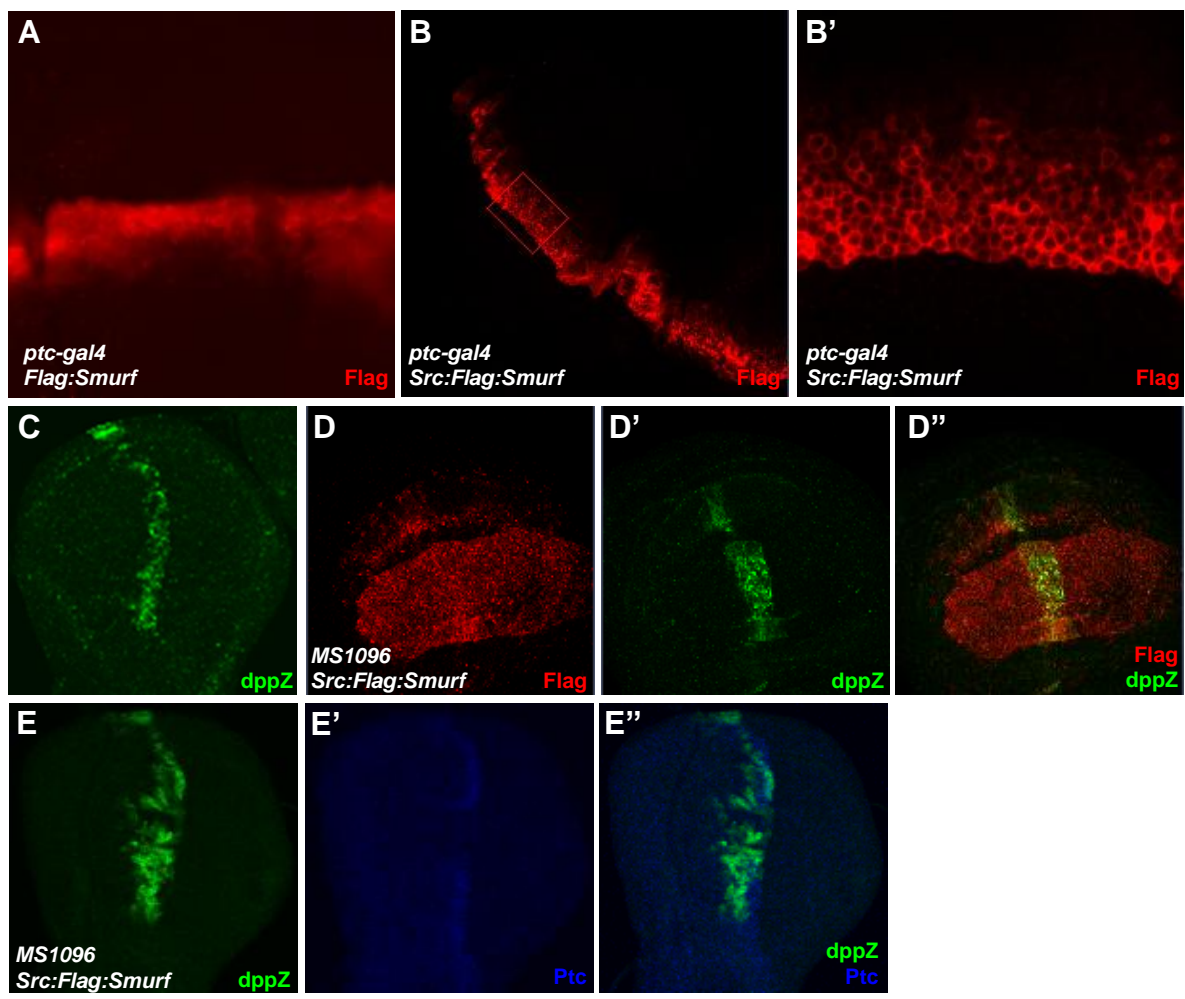

Supplement: Figure S6 — Membrane-tethered Smurf enhances its activity to regulate Hh signaling. (A–B′) Wing discs expressing Flag:Smurf (A) or Src:Flag-Smurf (B–B′) driven by ptc-gal4 were immunostained to show the localization of Smurf. (C) WT wing discs carrying dpp-LacZ (dppZ) reporter were immunostained with anti-β Gal antibody to show the expression of dppZ (green). (D–D″) Wing discs expressing Src:Flag:Smurf driven by MS1096-gal4 were immunostained to show the expression of dppZ (green) and Flag (red). (E–E″) Wing discs from early 3rd instar larva expressing Src:Flag:Smurf driven by MS1096-gal4 were immunostained to show the expression of dppZ (green) and Ptc (blue). (PDF) [file pbio.1001721.s006.pdf]

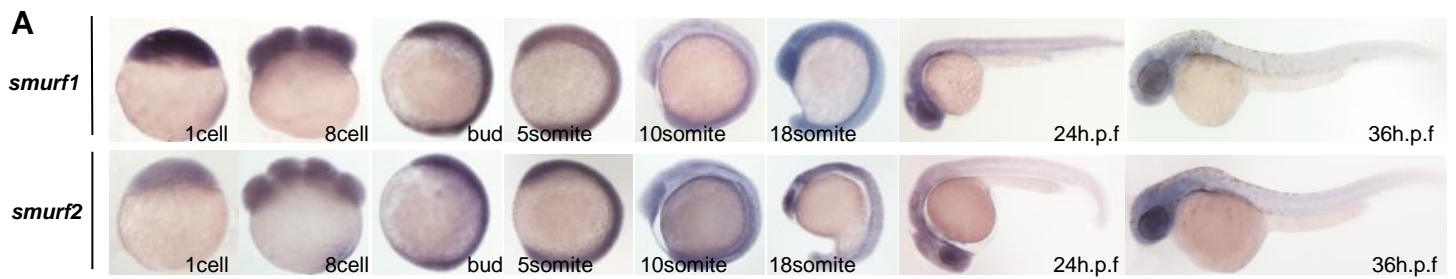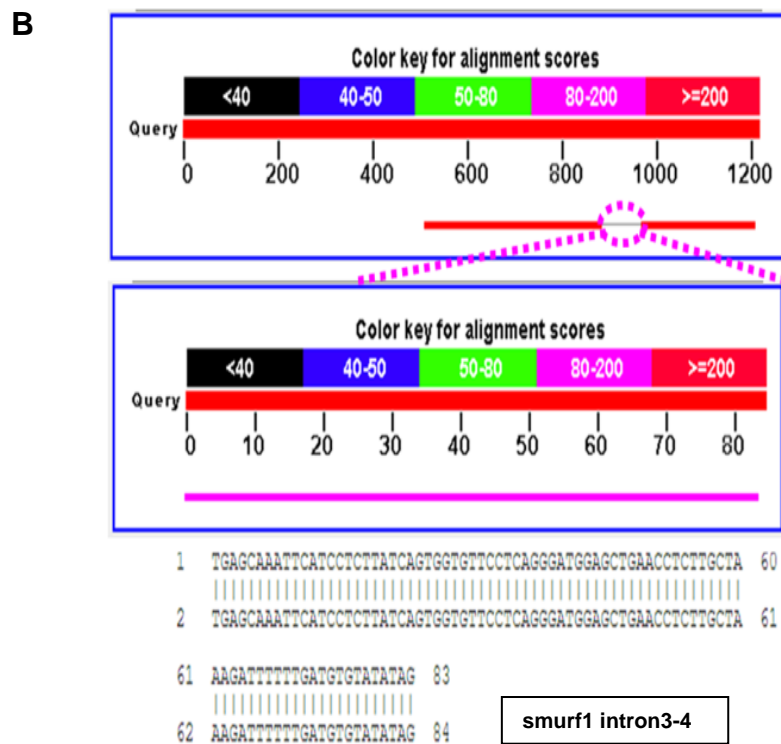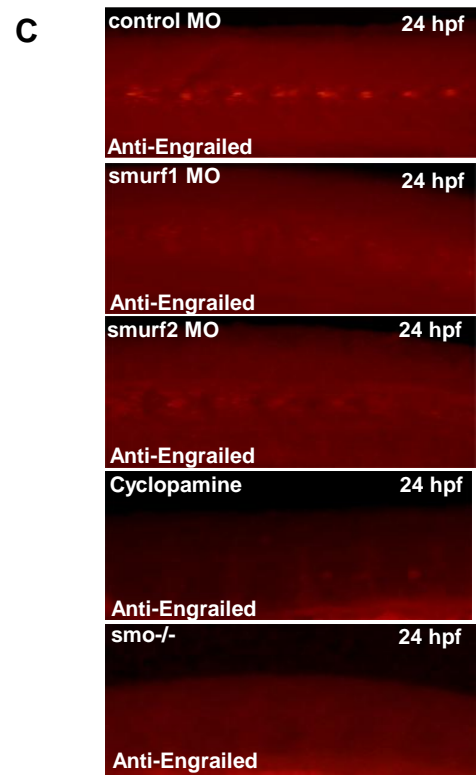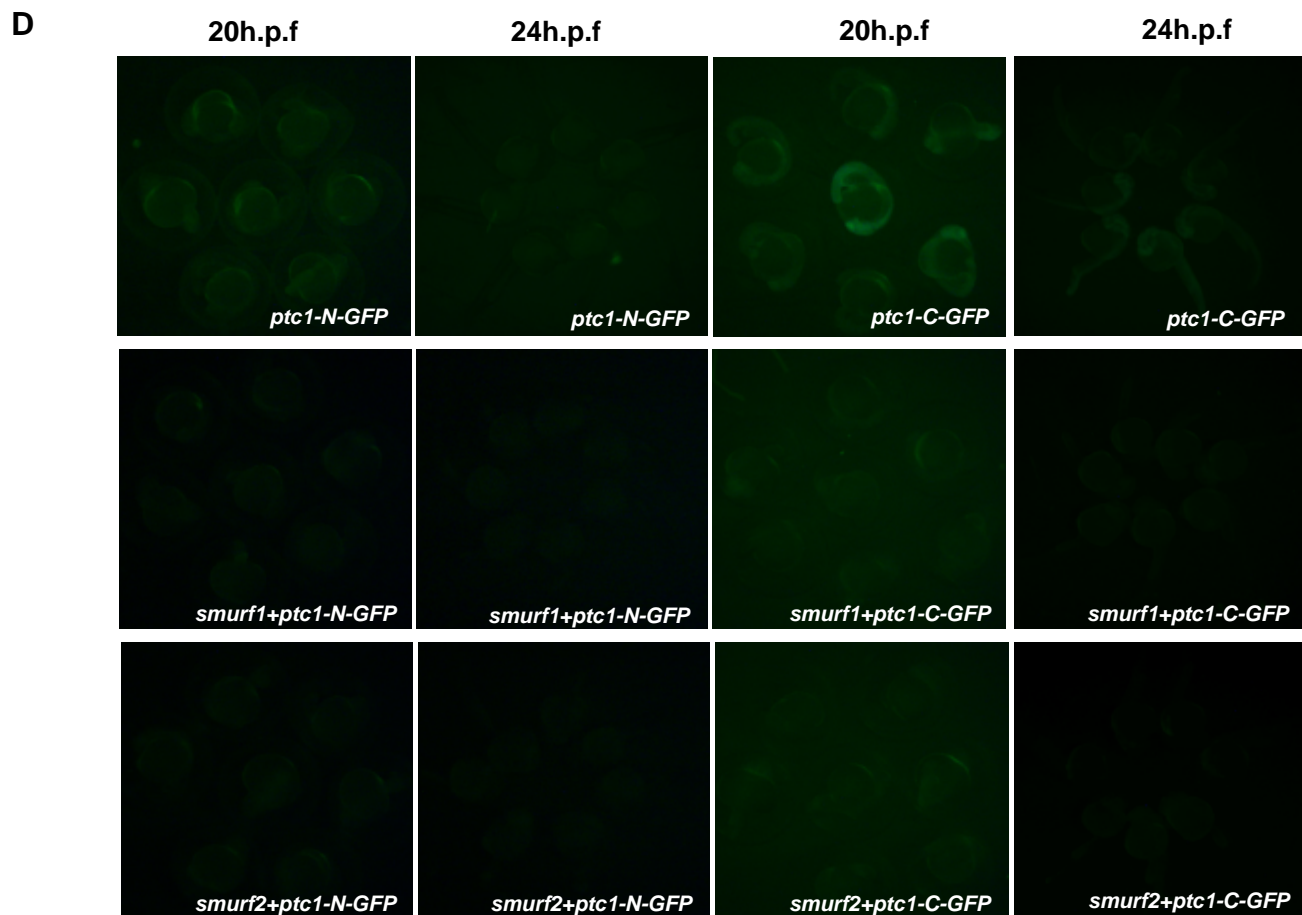

Supplement: Figure S7 — Expression patterns of smurf1 and smurf2 during zebrafish embryogenesis. Validation of smurf1 splicing MO, and degradation of Ptc:GFP fusion protein by Smurfs. (A) Both smurf1 and smurf2 mRNA are ubiquitously expressed in all stages of zebrafish embryonic development examined. (B) The specific effect of smurf1 splicing MO, which leads to retention of intron 3. (C) Downregulation of Hh-responsive target genes engrailed in smurf MOs. (D) Embryos injected with 100 pg ptc1-C-GFP mRNA show obvious GFP fluorescence at 20 hours post-fertilization (hpf) and 24 hpf. After co-injection of smurf mRNAs with ptc1-C-GFP mRNA, GFP expression was reduced. While embryos injected with 100 pg ptc1-N-GFP mRNA expressed weak GFP at 20 hpf and 24 hpf, there was no obvious change in embryos co-injected with smurf mRNAs and ptc1-N-GFP mRNA. (PDF) [file pbio.1001721.s007.pdf]

**A**

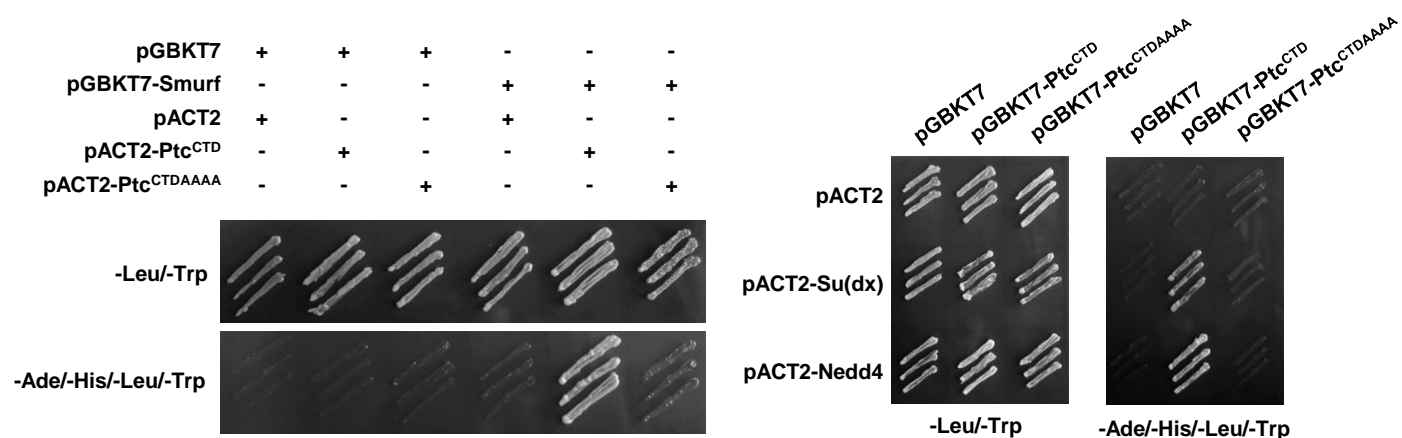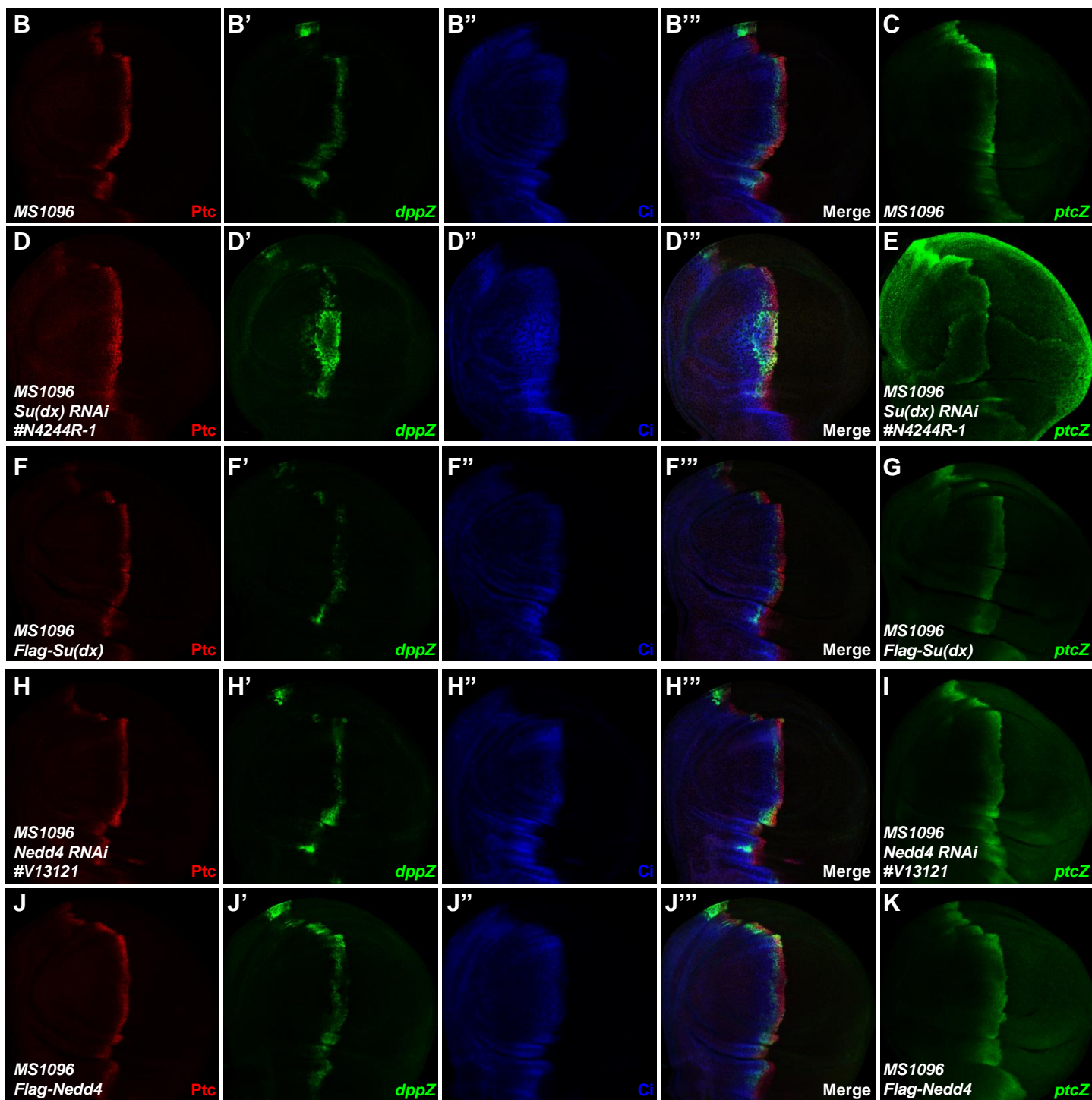

Supplement: Figure S8 — Nedd4 and Su(dx) interact with Ptc through the C-tail of Ptc, but have different role from Smurf in the regulation of Hh signaling. (A) Yeast two-hybrid was performed to show that the PPAY motif in PtcCTD is important for Ptc-Smurf direct interaction, and to show that Ptc directly interacts with Nedd4 and Su(dx). The AH109 yeast strain were transformed with the indicated plasmids and plated at permissive (−Leu/−Trp) and restrictive (−Ade/−His/−Leu/−Trp) medium. Full-length smurf and PtcCTD were cloned into the bait vector, pGBKT7, and pACT2 is the prey vector. (B–B′″) Control wing discs from MS1096-gal4 were immunostained to show the expression of Ptc (red), dppZ (green), and Ci (blue). (C) Control wing discs from MS1096-gal4 were immunostained to show the expression of ptcZ (green). (D–D′″) Wing discs expressing su(dx) knockdown transgene driven by MS1096-gal4 were immunostained to show the expression of Ptc (red), dppZ (green), and Ci (blue). (E) Wing discs expressing su(dx) knockdown transgene driven by MS1096-gal4 were immunostained to show the expression of ptcZ (green). (F–F′″) Wing discs expressing Flag:Su(dx) driven by MS1096-gal4 were immunostained to show the expression of Ptc (red), dppZ (green), and Ci (blue). (G) Wing discs expressing Flag:Su(dx) driven by MS1096-gal4 were immunostained to show the expression of ptcZ (green). (H–H′″) Wing discs expressing nedd4 knockdown transgene driven by MS1096-gal4 were immunostained to show the expression of Ptc (red), dppZ (green), and Ci (blue). (I) Wing discs expressing nedd4 knockdown transgene driven by MS1096-gal4 were immunostained to show the expression of ptcZ (green). (J–J′″) Wing discs expressing Flag:Nedd4 driven by MS1096-gal4 were immunostained to show the expression of Ptc (red), dppZ (green), and Ci (blue). (K) Wing discs expressing Flag:Nedd4 driven by MS1096-gal4 were immunostained to show the expression of ptcZ (green). (PDF) [file pbio.1001721.s008.pdf]

**A**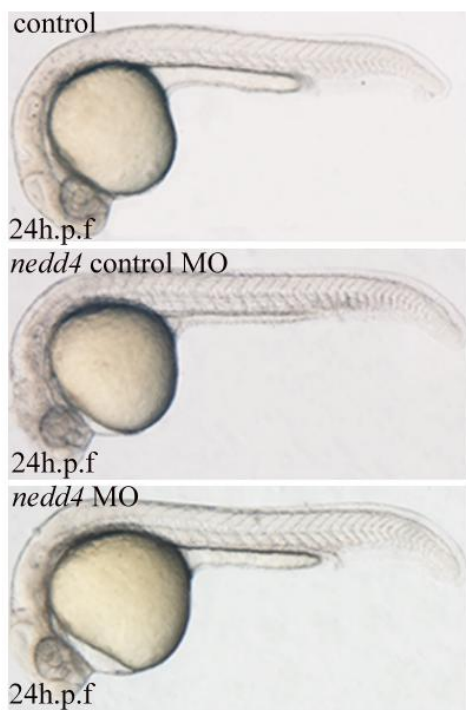**B**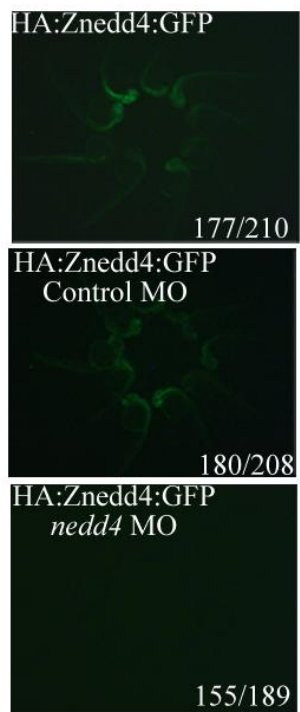**C**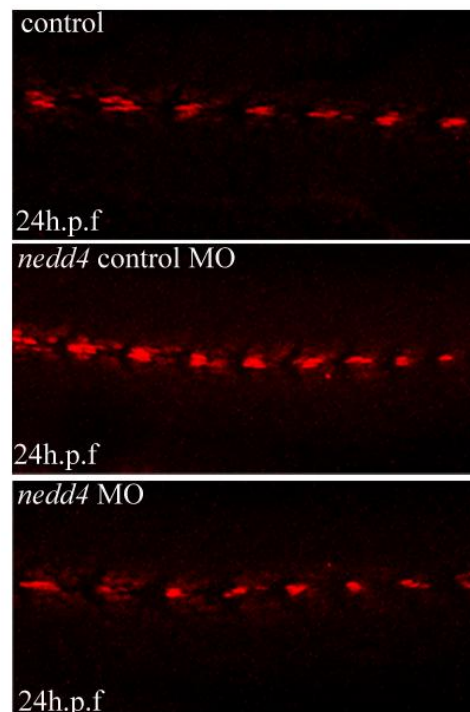**D**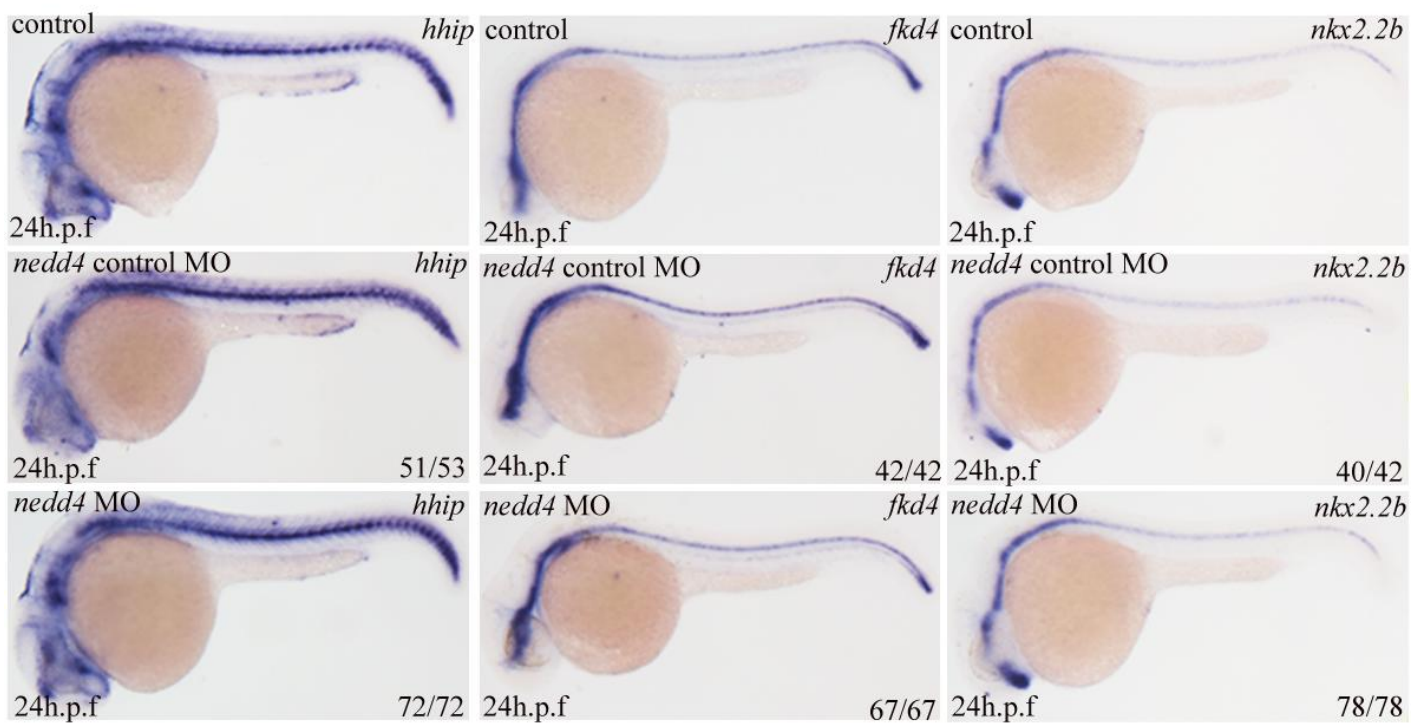

Supplement: Figure S9 — Zebrafish Nedd4 has no apparent role in regulating Hh signaling during embryonic development. (A) 1 ng nedd4 MO or control MO with equal dose of p53 MO was injected into the zebrafish one-cell stage embryos. The nedd4 morphants showed normal embryonic development, when compared with the control. (B) The mRNA of zebrafish nedd4 fused with a GFP tag was co-injected with 1 ng nedd4 MO or control MO and equal dose of p53 MO into the zebrafish one-cell stage embryos, nedd4 MO efficiently decreased GFP signal intensity. (C) 1 ng nedd4 MO or control MO with equal dose of p53 MO was injected into the zebrafish one-cell stage embryos. nedd4 morphants were immunostained with anti-En antibody to detect expression levels of Hh target gene En (red). (D) 1 ng nedd4 MO or control MO with equal dose of p53 MO was injected into the zebrafish one-cell stage embryos. In situ hybridization was performed for nedd4 morphants to detect expression levels of Hh target genes hhip, fkd4, and nkx2.2b. (PDF) [file pbio.1001721.s009.pdf]
