# Supplementary material for: Activation of Smurf E3 Ligase Promoted by Smoothened Regulates Hedgehog Signaling through Targeting Patched Turnover
Source: PLoS Biol. 2013 Nov 26;11(11):e1001721. doi: 10.1371/journal.pbio.1001721 (PMC3841102; doi:10.1371/journal.pbio.1001721)
Supplement: Figure S2 — Smurf regulates Ptc ubiquitination through its C-tail. (A) S2 cells were transfected with combinations of DNA constructs as indicated. After 48 h transfection, S2 cells were treated with MG132 (50 µM final concentration) and NH4Cl (50 mM final concentration) for 4 h. Cell lysates were then immunoprecipitated with mouse anti-Myc affinity gel. Western blotting was used to analyze the presence of indicated proteins and levels of ubiquitination of PtcCTD. (B and C) S2 cells were transfected with combinations of DNA constructs as indicated. After 48 h transfection, S2 cells were treated with MG132 (50 µM final concentration) and NH4Cl (50 mM final concentration) for 4 h. Harvested S2 cells were treated with denaturing buffer for 10 min and then immunoprecipitated with mouse anti-Myc affinity gel (B) or mouse anti-Flag affinity gel (C). Western blotting was performed to analyze the presence of indicated proteins and levels of ubiquitination of Ptc. (D) The panel shows the same Figure as Figure 2H, but a longer exposure time was used. (E) S2 cells were transfected with combinations of DNA constructs as indicated. After 48 h transfection, S2 cells were treated with MG132 (50 µM final concentration) and NH4Cl (50 mM final concentration) for 4 h. Harvested S2 cells were treated with denaturing buffer for 10 min and then immunoprecipitated with rabbit anti-HA antibody and protein A/G Sepharose beads. Western blotting was performed to analyze the presence of indicated proteins and levels of ubiquitination of Ptc. (F) S2 cells were transfected with combinations of DNA constructs as indicated. After 48 h transfection, lysates from transfected S2 cells were immunoprecipitated with anti-Flag M2 affinity gel. Western blots were performed to analyze the presence of Flag-tagged or Myc-tagged proteins. (G) An in vitro ubiquitination assay was performed according to the method in Text S1. After reaction, proteins were immuno-purified with anti-Ub antibody and Protein A/G Sepharose beads [file pbio.1001721.s002.pdf]

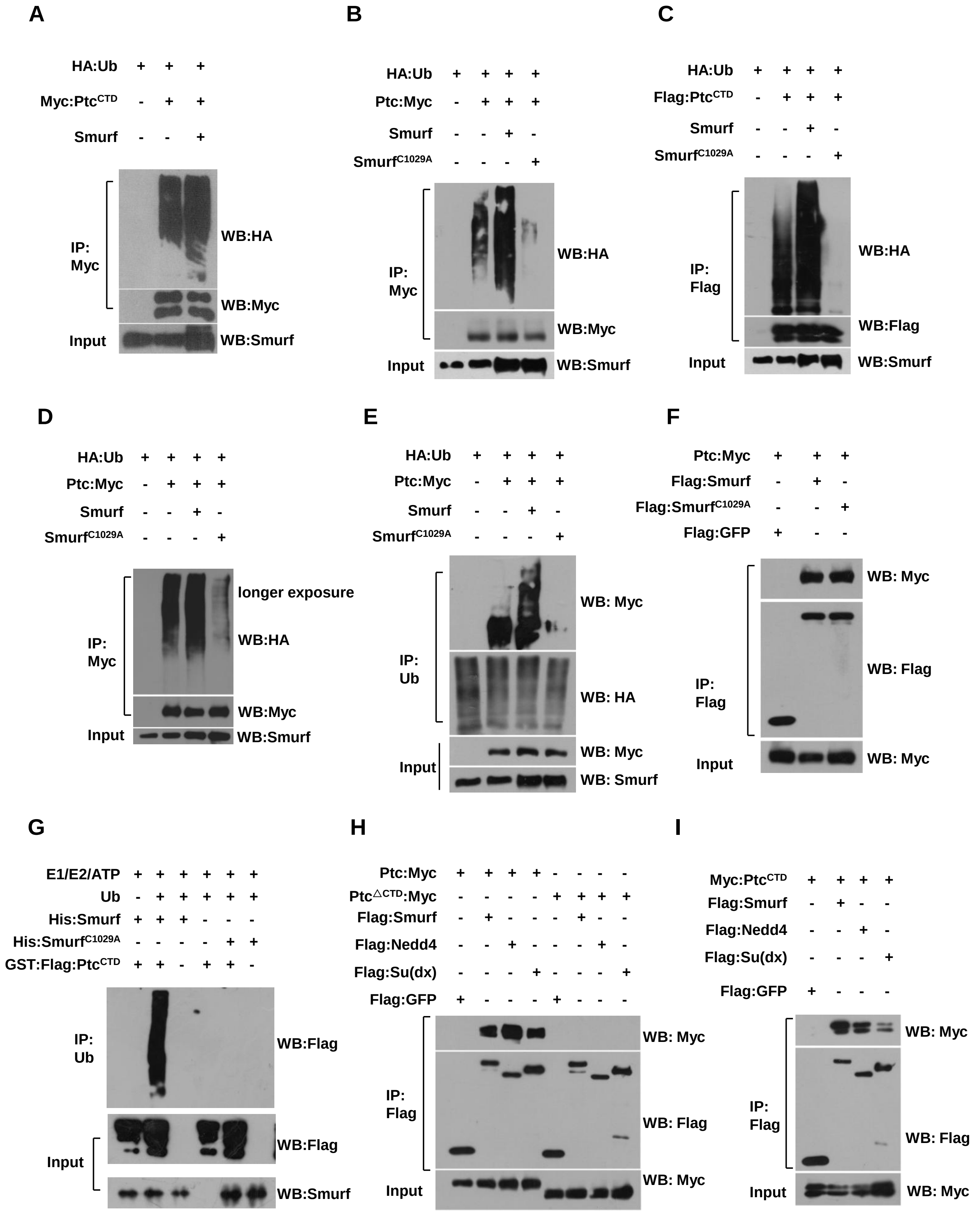
A
HA:Ub + + +
Myc:Ptc^CTD - + +
Smurf - - +
IP: Myc
WB:HA
WB:Myc
Input
WB:Smurf
B
HA:Ub + + + +
Ptc:Myc - + + +
Smurf - - + -
Smurf^C1029A - - - +
IP: Myc
WB:HA
WB:Myc
Input
WB:Smurf
C
HA:Ub + + + +
Flag:Ptc^CTD - + + +
Smurf - - + -
Smurf^C1029A - - - +
IP: Flag
WB:HA
WB:Flag
Input
WB:Smurf
D
HA:Ub + + + +
Ptc:Myc - + + +
Smurf - - + -
Smurf^C1029A - - - +
longer exposure
IP: Myc
WB:HA
WB:Myc
Input
WB:Smurf
E
HA:Ub + + + +
Ptc:Myc - + + +
Smurf - - + -
Smurf^C1029A - - - +
WB: Myc
IP: Ub
WB: HA
WB: Myc
Input
WB: Smurf
F
Ptc:Myc + + +
Flag:Smurf - + -
Flag:Smurf^C1029A - - +
Flag:GFP + - -
WB: Myc
IP: Flag
WB: Flag
Input
WB: Myc
G
E1/E2/ATP + + + + + +
Ub - + + + + +
His:Smurf + + + - - -
His:Smurf^C1029A - - - - + +
GST:Flag:Ptc^CTD + + - + + -
IP: Ub
WB:Flag
WB:Flag
Input
WB:Smurf
H
Ptc:Myc + + + + - - - -
Ptc^△CTD:Myc - - - - + + + +
Flag:Smurf - + - - - + - -
Flag:Nedd4 - - + - - - + -
Flag:Su(dx) - - - + - - - +
Flag:GFP + - - - + - - -
WB: Myc
IP: Flag
WB: Flag
Input
WB: Myc
I
Myc:Ptc^CTD + + + +
Flag:Smurf - + - -
Flag:Nedd4 - - + -
Flag:Su(dx) - - - +
Flag:GFP + - - -
WB: Myc
IP: Flag
WB: Flag
Input
WB: Myc
