# Supplementary material for: Activation of Smurf E3 Ligase Promoted by Smoothened Regulates Hedgehog Signaling through Targeting Patched Turnover
Source: PLoS Biol. 2013 Nov 26;11(11):e1001721. doi: 10.1371/journal.pbio.1001721 (PMC3841102; doi:10.1371/journal.pbio.1001721)
Supplement: Table S1 — Potential Smurf-interacting partners identified in a yeast two-hybrid screen. (PDF) [file pbio.1001721.s010.pdf]

Table S1. Potential Smurf-interacting partners identified in a yeast two-hybrid screen.

	Gene name	Hit counts	Molecular function
	CG9083	1	unknown
	Klp64D	1	Microtubule motor activity; ATP binding
	CG3731	1	Ubiquinol-cytochrome-c reductase activity and metalloendopeptidase activity
	CG3446	1	NADH dehydrogenase activity
	Ef2b	1	Translation elongation factor activity; GTP binding and GTPase activity
	ptc	3	Hedgehog receptor activity; transmembrane receptor activity and lipoprotein receptor activity
	CG10107	1	Cysteine-type peptidase activity
	CG10591	4	unknown
	CG7180	1	Protein tyrosine phosphatase activity
	ial	1	Protein kinase activity
	CG3016	1	Ubiquitin thiolesterase activity
	CG13630	1	Aminopeptidase activity and metalloexopeptidase activity
	Cp1	2	Cysteine-type endopeptidase activity
	obst-E	1	structural constituent of peritrophic membrane
	CG32499	1	chitin binding
	CG32302	1	chitin binding
	CG34126	1	nucleotide binding
	Rbsn-5	1	zinc ion binding
	raw	1	unknown
	Zyx	3	zinc ion binding
	rump	4	mRNA 3'-UTR binding; mRNA binding and nucleotide binding
	CG11652	1	unknown
	Trp1	1	protein transporter activity
	CG10990	1	unknown
	Traf4	1	protein binding; zinc ion binding
	Rpt4	1	ATPase activity; endopeptidase activity and ATP binding
	Nc73EF	1	oxoglutarate dehydrogenase (succinyl-transferring) activity
	alc	1	AMP-activated protein kinase activity
	RpL18A	1	structural constituent of ribosome

	Pax	1	zinc ion binding
	CG5708	1	zinc ion binding
	CG6522	1	zinc ion binding
	ade5	1	Phosphoribosylaminoimidazole carboxylase activity; Phosphoribosylaminoimidazolesuccinocarboxamide synthase activity and ATP binding
	CG30118	1	unknown
	Sdic2	1	unknown
	Rp L24	1	structural constituent of ribosome
