# Supplementary material for: Activation of Smurf E3 Ligase Promoted by Smoothened Regulates Hedgehog Signaling through Targeting Patched Turnover
Source: PLoS Biol. 2013 Nov 26;11(11):e1001721. doi: 10.1371/journal.pbio.1001721 (PMC3841102; doi:10.1371/journal.pbio.1001721)
Supplement: Table S2 — Parameters in Equation 1 . (PDF) [file pbio.1001721.s011.pdf]

Table S2. Parameters in Eq. 1.

**Parameter**	**Description**	**Value**	**Reference**
C_{Hh}	Synthesis rate of Hh	$10^{-4}\,\mu M\cdot s^{-1}$	Estimation
α_{Hh_Ptc}	Association rate of Hh_Ptc complex	$7.15\times10^{-2}\ \mu M^{-1}s^{-1}$	Lander et al. (2002) for Dpp
α_{Ptc}	Maximum synthesis rate of Ptc, which is given by $\alpha_{Ptc} = T_{Ptc}\alpha_{ptc}/\beta_{ptc}$, where $T_{Ptc} = 3.6\times10^{-3}\ s^{-1}$, $\alpha_{ptc} = 2.7\times10^{-5}\ \mu M^{-1}s^{-1}$, $\beta_{ptc} = 1.4\times10^{-4}\ s^{-1}$.	$6.94\times10^{-4}\,\mu M^{-1}s^{-1}$	Nahmad and Stathopoulos (2009)
$\hat{\alpha}_{Ptc}$	Fundamental synthesis rate of Ptc, which is given by $\hat{\alpha}_{Ptc} = T_{Ptc}\hat{\alpha}_{ptc}/\beta_{ptc}$, where $\hat{\alpha}_{ptc} = 3.8\times10^{-6}\ \mu M^{-1}s^{-1}$.	$9.77\times10^{-5}\,\mu M^{-1}s^{-1}$	Nahmad and Stathopoulos (2009)
$\alpha_{Smo/Ci}$	Maximal synthesis rate of activated Smo/Ci	$1.6\times10^{-4}\,\mu M^{-1}s^{-1}$	Denef et al. (2000)
α_{Smurf}	Maximal synthesis rate of activated Smurf	$4\times10^{-2}\,\mu M^{-1}s^{-1}$	Estimation
k_{ptc}	mRNA *ptc* half-maximal activation conc.	$0.14\ \mu M$	Nahmad and Stathopoulos (2009)
$k_{Smo/Ci}$	Smo/Ci half-maximal activation	2.135	Casali and Struhl (2004)
k_{Smurf}	Smurf half-maximal activation	3	Estimation
β_{Hh}	Hh degradation rate	$3\times10^{-6}\,s^{-1}$	Estimation
β_{Ptc}	Ptc maximal degradation rate	$1.5\times10^{-2}\ s^{-1}$	French and Lauffenburger (1996)
$\hat{\beta}_{Ptc}$	Ptc fundamental degradation rate	10^{-5}	Estimation
β_{Hh_Ptc}	Hh_Ptc degradation rate	$10^{-4}\,s^{-1}$	Estimation

$\beta_{Smo/Ci}$	Activated Smo/Ci degradation rate	$5.5\times10^{-4}\ s^{-1}$	Nahmad and Stathopoulos (2009)
β_{Smurf}	Activated Smurf degradation rate	$3\times10^{-4}s^{-1}$	Estimation
$\tilde{k}_{Ptc}$	Ptc half-maximal degradation	1	Estimation
m	Hill coefficient (*ptc* activation)	3	Eldar et al. (2003)
n	Hill coefficient (Smo/Ci activation)	6.8	Casali and Struhl (2004)
θ	Hill coefficient (Ptc degradation)	2	Estimation
ϑ	Hill coefficient (Smurf activation)	2	Estimation
