# Supplementary material for: Activation of Smurf E3 Ligase Promoted by Smoothened Regulates Hedgehog Signaling through Targeting Patched Turnover
Source: PLoS Biol. 2013 Nov 26;11(11):e1001721. doi: 10.1371/journal.pbio.1001721 (PMC3841102; doi:10.1371/journal.pbio.1001721)
Supplement: Text S1 — Supplemental materials and methods. (DOCX) [file pbio.1001721.s012.docx]

**Text S1, Supplemental materials and methods**

**Supplemental data for mathematic modeling**

**Stability analysis of Eq. 1**

In order to determine the rest point of the system, denoted by $\mathbf{x}*=\left(x_{1}^{*},x_{2}^{*},x_{3}^{*},x_{4}^{*},x_{5}^{*}\right)$

Notice that

$$\frac{dx_{1}}{dt}=0\text{ }\to\text{ }x_{1}=\frac{C_{Hh}}{\alpha_{Hh\_Ptc}x_{2}+\beta_{Hh}}$$

$$\frac{dx_{4}}{dt}=0\text{ }\to\text{ }x_{4}=\frac{1}{\beta_{Smo/Ci}}\cdot\frac{\alpha_{Smo/Sci}\left(x_{3}/x_{2}\right)^{n}}{k_{Smo/Ci}^{n}+\left(x_{3}/x_{2}\right)^{n}}$$

$$\frac{dx_{5}}{dt}=0\text{ }\to\text{ }x_{5}=\frac{1}{\beta_{Smurf}}\cdot\left[\frac{\alpha_{Smurf}x_{4}^{\vartheta}}{k_{Smurf}^{\vartheta}+x_{4}^{\vartheta}}+\hat{\alpha}_{Smurf}\right]$$

Thus, the equilibrium of the system can be determined by the following equation

$$\begin{matrix} \frac{dx_{2}}{dt}=0\text{ }\to\text{ }\frac{\alpha_{Ptc}x_{4}^{m}}{k_{ptc}^{m}+x_{4}^{m}}+\hat{\alpha}_{Ptc}-\frac{C_{Hh}\alpha_{Hh\_Ptc}}{\alpha_{Hh\_Ptc}x_{2}+\beta_{Hh}}x_{2}-\left[\frac{\beta_{Ptc}x_{5}^{\theta}}{\tilde{k}_{Ptc}^{\theta}+x_{5}^{\theta}}+\hat{\beta}_{Ptc}\right]x_{2}=0 \\ \frac{dx_{3}}{dt}=0\text{ }\to\text{ }\frac{C_{Hh}\alpha_{Hh\_Ptc}}{\alpha_{Hh\_Ptc}x_{2}+\beta_{Hh}}x_{2}-\beta_{Hh\_Ptc}x_{3}=0 \end{matrix}$$

where $\text{ }x_{5}=\frac{1}{\beta_{Smurf}}\cdot\left[\frac{\alpha_{Smurf}x_{4}^{\vartheta}}{k_{Smurf}^{\vartheta}+x_{4}^{\vartheta}}+\hat{\alpha}_{Smurf}\right]$ and .

For given parameters in Eq. 2, which are defined in Table S1, the numerical analysis shows that the fixed point is unique. From the characteristic equation of the Jacobian matrix about $\mathbf{x}*$ (using the Hurwitz criterion), the rest point $\mathbf{x}*$ is asymptotically stable and it is also globally stable under the given parameter values.

**Supplemental Experimental procedures**

**Zebrafish strains and morpholino knockdown in zebrafish**

Zebrafish (*Danio rerio*) embryos were derived from the Tübingen strain. Embryos were incubated in Holtfreter’s solution at 28.5 °C and staged. Antisense morpholino oligonucleotides (MOs) were designed against the start codon 5′ untranslated region to block translation and control morpholino oligonucleotides (CMOs) were designed using mismatched oligonucleotides.

MO and CMO sequences were as follows:

*smurf1*-MO: 5´-AGTCCCAGGATTCGACATCACTCCC-3´

*smurf1* splice MO: 5´-AAGAGGATGAATTTGCTCACAGGTC-3´

*smurf2*-MO: 5´-CATGTCCTTGACCCTTATCCAGGCT-3´

*smurf1*-CMO: 5´-AGTCCCAGTCGAGTACATCACTCCC-3´

*smurf2*-CMO: 5´-CATGTCCTGACACCTTATCCAGGCT-3´

*patched1*-MO : 5´-CATAGTCCAAACGGGAGGCAGAAGA -3´

*nedd4*-MO: 5´-CGGGAGTTGCGCGGCCATGTTCAT-3´

*nedd4*-CMO: 5´-CGGGAGTTGCGCTAGCGCGTTCAT-3´

Morpholinos were injected at a concentration of 0.5mM, except Ptc1 MO, which was injected at 0.4 mM. Embryos were analyzed morphologically at 24 hours post fertilization (hpf) after injection.

**Constructs for mRNA in vitro transcription and mRNA microinjection**

The full length or fragment DNAs of zebrafish *Smurf1,* *smurf2* and *patched1* were amplified by PCR using the following oligonucleotides, and RNAs were generated for embryo microinjection

*smurf1*:

F: 5´- ATGTCGAATCCTGGGACT-3´

R: 5´- TCACTCCACTGCGAAACCACATG -3´

*smurf2*:

F: 5´- ATGGACCCGTTAAGTTGCGTTT-3´

R: 5´- TCATTCCACAGCGAAGCCAC-3´

*patched1*:

F: 5´- ATGGCCTCGGCTGTTAATGT-3´

R: 5´- TCAGCTGGAGGGTCTTGTGT-3´

*patched1*-N:

F: 5´-ATGGCCTCGGCTGTTAATGT-3´

R: 5´-TCCGCTGAGGAATCGGCATAC -3

*patched1*-C:

F: 5´- ATG CGCTACAGCCCTCCACCCTCC -3´

R: 5´-CC TCTGCTGGAGGGTCTT -3´

Full-length *smurf1* and *smurf2* cDNAs were sub-cloned into kpn1 and spe1 sites on the pXT7 vector containing the T7 promoter.

The *patched1*-N and *patched1*-C were subcloned into kpn1/ xho1 and xho1/AflII sites respectively, and were fused with GFP on the pXTG3 vector which containing T7 promoter. The mRNAs were synthesized in vitro with the mMESSAGE mMACHINE Kit (Ambion). An RNeasy Mini Kit (QIAGEN) was used for mRNA purification.  The mRNAs were then injected into MO-injected or wild-type embryos at the one-cell stage at concentrations of 100 ng/µl.

**NCBI Reference Sequence:**

Zebrafish smurf1: NM_001001943.1

Zebrafish smurf2: NM_001114426.1

Zebrafish patched1: XM_001922126.4

**In situ hybridization and immunofluorescence staining**

Digoxigenin (DIG)-labeled antisense RNA probes were synthesized using the DIG RNA labeling kit (Roche). Whole-mount *in situ* hybridization was carried out by the following standard procedures. We performed mRNA probe synthesis and whole-mount in situ hybridization as described previously [1]. The probes were amplified using the following oligonucleotides:

*hhip*:

F: 5’-ctccatacccagaaacaatccc-3’

R: 5’-tgaagagcgagtcagcttcc-3’

*fkd4*:

F: 5’-atattctcccccagagcatc-3’

R: 5’- tttccactttcacatcttctcc-3’

*nkx2.2b*:

F: 5’-agaaaaaacagcacgactcc-3’

R: 5’-aatccaccttgcagtaaagac-3’

Whole-mount immunofluorescence staining on zebrafish embryos was performed according to methods described previously [2]. The anti-F59 and anti-EN antibodies were used: 1:100 dilution.

**Supplemental *In vivo* Ubiquitination Assay**

In addition to the method shown in the main text, we also used the following methods to perform in vivo ubiquitination assay for Ptc.

1. The method was carried out according to the previous paper [3,4]. S2 cells were transfected with indicated constructs, at 48 h posttransfection, cells were treated with MG132 (at a final concentration of 50 µM) and/or NH_4_Cl (at a final concentration of 50 mM) for 4 h. harvested cells were then lysed with denaturing buffer (1% SDS, 50 mM Tris, pH 7.5, 0.5 mM EDTA, and 1 mM DTT) and incubated at 100°C for 10 min to destroy protein native structure and any non-covalent protein-protein interactions. The lysates were then diluted 5-fold with regular lysis buffer containing 1.5 mM MgCl_2_ to decrease the SDS concentration and subjected to immunoprecipitation with an anti-Myc antibody or anti-M2 Flag affinity gel (Sigma). After pull-down for 4h, the beads were then extensively washed with lysis buffer containing 0.1% SDS and 500 mM NaCl three times for a total 1h. Samples were then subjected to western blot analysis with anti-Ub antibody or anti-HA antibody.

2. S2 cells were transfected, lysed and boiled in denaturing buffer using the same method above. However, we used anti-Ub antibody (or anti-HA antibody) to immunoprecipitate ubiquitinated protein, and then used anti-Myc antibody to detect the levels of Ptc ubiquitination by performing western blot assays.

***In vitro* Ubiquitination Assay of Ptc^CTD^**

For the in vitro ubiquitination assay, GST:Flag:Ptc^CTD^ substrate protein was bacterially expressed and purified by glutathione agarose beads. E3 ligase His:Smurf^WT^ and His:Smurf^C1029A^ were bacterially expressed and purified by Ni Sepharose beads. In vitro ubiquitination was performed in buffer containing 50 mM Tris-HCl ,pH 7.5, 1 mM DTT, 50 mM NaCl, 5 mM MgCl_2_, and 2 mM ATP. 50 ng E1, 150 ng E2 (UbcH5C), 500 ng His:Smurf E3 ligase, 1 ug substrate and 10 ug Ub proteins were used for each reaction system at 30°C for 2 hr in a total of 40 ul reaction volume. Reactions were stopped and purified with anti-Ub antibody plus protein A/G beads and analyzed by western blotting with anti-Flag antibody to show levels of ubiquitination of Ptc^CTD^.

**Supplemental References**

1. Xia L, Jia S, Huang S, Wang H, Zhu Y, et al. (2010) The Fused/Smurf complex controls the fate of Drosophila germline stem cells by generating a gradient BMP response. Cell 143: 978-990.

2. Ma D, Wang L, Wang S, Gao Y, Wei Y, et al. (2012) Foxn1 maintains thymic epithelial cells to support T-cell development via mcm2 in zebrafish. Proc Natl Acad Sci U S A 109: 21040-21045.

3. Xia R, Jia H, Fan J, Liu Y, Jia J (2012) USP8 promotes smoothened signaling by preventing its ubiquitination and changing its subcellular localization. PLoS Biol 10: e1001238.

4. Li S, Chen Y, Shi Q, Yue T, Wang B, et al. (2012) Hedgehog-regulated ubiquitination controls smoothened trafficking and cell surface expression in Drosophila. PLoS Biol 10: e1001239.
